# An Economic Scheduling Management Method for Microgrids Using Multi-Strategy Improved Sand Cat Swarm Optimization

**DOI:** 10.3390/biomimetics10110735

**Published:** 2025-11-02

**Authors:** Bingnan Liu, Zhiyi Song, Huiji Wang

**Affiliations:** 1 School of Business, Macau University of Science and Technology, Taipa, Macau 999078, China; 3220006448@student.must.edu.mo; 2 School of Automation, Beijing Information Science and Technology University, No. 55 Taihang Road, Changping District, Beijing 102206, China; 2023010772@bistu.edu.cn; 3 School of Humanities and Public Administration, Jiangxi Agricultural University, Nanchang 330045, China

**Keywords:** microgrid scheduling, intelligent optimization algorithm, sand cat swarm optimization, IEEE CEC2017

## Abstract

With the rise of the digital economy, energy management has become increasingly intelligent and data-driven. Environmental, Social, and Governance (ESG) considerations have emerged as a key driver of corporate competitiveness, while microgrid scheduling serves as an essential pathway for enterprises to achieve carbon reduction, attract green investment, and meet low-carbon development goals. However, traditional microgrid economic dispatch algorithms often suffer from low optimization efficiency, limited scalability, and poor flexibility. To address these challenges, this paper proposes a multi-strategy improved sand cat swarm optimization (MISCSO) algorithm for the economic scheduling of microgrids. First, a distribution-optimized initialization method based on adaptive diversity guidance is developed to enhance the quality of the initial population. This approach improves algorithmic performance by generating individuals in high-potential regions to ensure solution quality while maintaining population diversity through the inclusion of individuals from low-potential regions. Subsequently, an elite-centered global random movement strategy is introduced to balance elite guidance and global exploration, thereby improving both convergence speed and optimization accuracy. In addition, an adaptive elastic boundary mapping mechanism is proposed to effectively handle boundary violations, striking a balance between boundary constraints and global search capability. To evaluate the effectiveness of MISCSO, it is compared with 11 state-of-the-art algorithms using the IEEE CEC2017 benchmark set, and statistical analyses are conducted to assess performance differences. Experimental results demonstrate that MISCSO achieves superior optimization accuracy, convergence performance, and robustness. Finally, the applicability of MISCSO is verified through its implementation in microgrid economic scheduling, where it achieves outstanding optimization results.

## 1. Introduction

ESG (Environmental, Social, and Governance) has become a key competitive advantage for enterprises (such as attracting green investments and meeting customers’ low-carbon requirements), and microgrid dispatch serves as a critical means for enterprises to achieve their “carbon reduction” goals. In this process, microgrid dispatch serves as an efficient and flexible energy management tool, providing viable technical support for enterprises to achieve low-carbon operational goals [1]. By optimizing dispatch strategies, enterprises can better integrate distributed renewable energy, energy storage, and load demands, significantly reducing carbon emissions while ensuring energy supply security and economic efficiency. Simultaneously, microgrid dispatch enhances transparency and controllability in energy usage, providing data support for corporate ESG disclosure and green audits. This not only aids enterprises in achieving carbon reduction targets but also strengthens their competitive edge in green investment markets and international supply chains.

Moreover, the current era of limited energy resources demands higher standards for efficient energy management and intelligent decision-making. As an integrated platform that combines distributed energy systems with digital management technologies, research on microgrid economic dispatch not only enables enterprises to achieve their “carbon reduction” goals but also supports enhanced decision-making and operational efficiency through data-driven approaches. From a management perspective, microgrid economic dispatch provides a methodological foundation for the digital transformation of energy management while offering practical support for industrial optimization and value creation within the digital economy. Consequently, researching microgrid economic dispatch holds significant importance for advancing intelligent energy management and enhancing operational efficiency in the digital economy.

Existing microgrid economic dispatch algorithms can be primarily categorized into traditional optimization algorithms [2], heuristic algorithms [3], and data-driven approaches [4]. Traditional optimization algorithms include linear programming and mixed-integer linear programming. These algorithms typically rely on precise mathematical models and impose stringent requirements on system characteristics and constraints. However, the high variability of renewable energy sources and significant load uncertainty in actual microgrids often make it difficult to fully satisfy the model assumptions. Artificial intelligence and data-driven approaches encompass methods like reinforcement learning and deep reinforcement learning. These methods heavily depend on high-quality data, exhibit poor interpretability, involve high training and computational costs, and possess limited generalization capabilities. Additionally, integrating them into practical management systems presents significant challenges.

In contrast, intelligent optimization algorithms leverage robust global search capabilities, adaptability to nonlinear problems, multi-objective optimization capabilities, and real-time adaptive scheduling. They not only enhance the economic viability and operational efficiency of microgrids but also fully utilize data resources to provide intelligent support for energy management and corporate decision-making within the digital economy [5]. Therefore, the application of intelligent optimization algorithms in microgrid economic dispatch balances theoretical feasibility, computational efficiency, and practical management value. It has emerged as a key methodology for advancing digital energy management, leading an increasing number of researchers to adopt intelligent optimization algorithms to address microgrid economic dispatch challenges.

For example, Wang et al. established an economic dispatch model for microgrids by integrating electric vehicles with microgrid systems, aiming to minimize operational costs and pollutant treatment expenses [6]. They proposed a multi-strategy real-valued evolutionary algorithm (RVEA) to enhance the economic and environmental protection of microgrid systems, thereby promoting their optimized operation. To provide an uninterruptible power supply with improved voltage regulation (VR), Selvaraj et al. proposed a novel method for optimizing power dispatch in microgrids based on the Raven Search Algorithm [7], specifically for effective load scheduling within distribution systems. Experimental results demonstrate that the Raven Search Algorithm significantly optimizes load scheduling, thereby substantially reducing power losses and enhancing voltage profiles across all scenarios. To achieve efficient optimization dispatch of microgrid cluster (MGC) systems while ensuring the safe and stable operation of the power grid, Wang et al. developed a multi-objective coordinated optimization fitness function based on operational costs, environmental costs, and energy storage losses [8]. They proposed an improved GWO (CDGWO) algorithm for optimization, effectively realizing multi-objective coordinated optimization dispatch of MGC systems. This approach significantly enhances the overall economic efficiency of MGCs while guaranteeing a reliable power supply.

However, as the No Free Lunch Theorem (NFL) states, no single algorithm can solve all optimization problems. Researchers must tailor and refine algorithms to address their specific challenges, ensuring optimal performance for their particular problems. For instance, Xie et al. proposed three strategies to enhance the rime optimization algorithm based on their specific three-dimensional drone path planning problem [9]. They demonstrated the effectiveness of these improvements through three test datasets and ultimately applied them to drone path planning. Compared to the original RIME algorithm, their approach yielded significantly better results. To address multi-level image segmentation challenges, Yue et al. integrated the Invasive Weed Optimization algorithm (IWO) and Grasshopper Optimization Algorithm (GOA) [10], enhancing search precision and accelerating convergence rates. By incorporating a random walk strategy, they further improved algorithm performance, achieving excellent results in image segmentation tasks. Yue et al. proposed a Monarch Butterfly Algorithm based on particle swarm optimization to enhance coverage in wireless sensor networks (WSNs) [11]. This approach optimizes energy consumption, operational performance, and coverage range. Experimental results demonstrate that the proposed improved method effectively reduces network costs while enhancing network coverage and node utilization more efficiently than the original single method.

The sand cat swarm optimization is a nature-inspired optimization algorithm [12]. Inspired by the Sand Cat’s ability to detect low frequencies below 2 kHz and its remarkable skill in digging out prey, it optimizes problems by simulating these two behaviors. This approach enables the discovery of good solutions with fewer parameters. Experiments demonstrate its outstanding performance in problems such as welding beam design and tension/compression spring design. Furthermore, despite SCSO’s already solid performance, it inevitably exhibits certain shortcomings when addressing problems across various domains. This necessitates targeted improvements by researchers to better tailor it for solving problems specific to each field. For instance, Yao et al. proposed three improvement strategies based on SCSO to address their specific feature selection problem: a novel adversarial learning strategy, a new exploration mechanism, and a biological elimination update mechanism [13]. Experiments demonstrated that the proposed algorithm performed better in feature selection tasks, achieving strong optimization capabilities. To address path planning for unmanned aerial vehicles (UAVs) in complex environments with multiple threats, Zhan et al. proposed an improved SCSO algorithm that integrates chaotic mapping initialization with a simulated annealing–particle swarm hybrid development strategy [14]. Experimental results demonstrate that the proposed strategy not only significantly enhances convergence speed while maintaining algorithmic accuracy but also provides an effective approach for improving SCSO performance.

Therefore, to address the problem of economic dispatch in microgrids, based on the above research, this paper proposes a multi-strategy improved SCSO (MISCSO). The specific contributions are as follows:

1. To enhance the quality of the initial population, a distribution optimization initialization based on adaptive diversity guidance is proposed. This method enhances algorithm performance by generating individuals in high-potential regions to ensure initial solution quality, while also generating individuals in low-potential regions to maintain population diversity.
2. We proposed an elite-centered global random movement strategy that balances elite guidance and global diversity, enhancing both the convergence speed and accuracy of the algorithm.
3. We propose an adaptive elastic boundary mapping method to effectively handle out-of-bound individuals, balancing boundary constraints with enhanced global search capabilities to improve algorithm performance.
4. The algorithms were qualitatively analyzed using 30 test functions from the IEEE CEC2017 test set and compared with 11 other algorithms to obtain competitive results. Most importantly, the algorithms were statistically analyzed to fully analyze the superior performance of MISCSO.
5. The MISCSO is applied to the problem of economic dispatch in microgrids and compared with other comparative algorithms.

The next part of this paper is organized as follows: Section 2 gives a brief introduction of the SCSO; Section 3 details the three improved strategies proposed in this paper for addressing the problem of economic dispatch in microgrids; in Section 4, we apply the MISCSO in numerical optimization experiments and analyze the experimental results in detail; in Section 5, we apply the algorithm to the problem of economic dispatch in microgrids and provide a comprehensive analysis of its advantages and disadvantages; and in Section 6, we summarize and provide an outlook on the work in this paper to clarify the direction of future work.

## 2. Sand Cat Swarm Optimization (SCSO)

Since the economic dispatch management algorithm for microgrids presented in this paper is an enhanced version of SCSO, this section provides a brief introduction to the original SCSO algorithm. Detailed specifications are as follows:

### 2.1. Initial Population

When solving optimization problems, we need to define the values of relevant variables based on the specific problem being optimized. For example, in particle swarm optimization, population individuals are referred to as particle positions; in gray wolf optimizer, they are called gray wolf positions; and in SCSO, they are termed sand cat positions. Like most optimization algorithms, SCSO employs a random initialization method to obtain the initial population. This involves randomly generating individuals within the problem’s search space, with the initialization formula shown in Equation (1).

(1) $$D={\left[\begin{array}{c} D_{1}\\ \vdots \\ D_{i}\\ \vdots \\ D_{N} \end{array}\right]}_{N\times dim}={\left[\begin{array}{ccccc} d_{1,1} & \ldots & d_{1,j} & \ldots & d_{1,dim}\\ \vdots & \ddots & \vdots & \ddots & \vdots \\ d_{i,1} & \ldots & d_{i,j} & \ldots & d_{i,dim}\\ \vdots & \ddots & \vdots & \ddots & \vdots \\ d_{N,1} & \ldots & d_{N,j} & \ldots & d_{N,dim} \end{array}\right]}_{N\times dim},$$

where D denotes the initial population of SCSO, $D_{i}$ denotes the i-th sand cat, N denotes the population size, and m denotes the problem dimension. $d_{i,j}$ can be calculated using Equation (2).

(2) $$d_{i,j}={lb}_{j}+rand\times ({ub}_{j}-{lb}_{j}),$$

where ${ub}_{j}$ and ${lb}_{j}$ represent the upper and lower bounds of the optimization problem, respectively, while rand denotes a random number between 0 and 1.

### 2.2. Searching the Prey (Exploration)

After initializing the sand cat population, the sand cats must search for prey to sustain their daily lives. At this stage, sand cats rely on low-frequency noise emission to locate prey. To simulate this behavior in mathematical modeling, Amir Seyyedabbasi et al. modeled low-frequency noise as a parameter linearly decreasing from 2 to 0 [12]. This indicates that sand cats exhibit peak sensitivity to prey detection during the early algorithmic phase, with sensitivity gradually diminishing as they approach their target. The variation in low-frequency noise ($r_{G})$ with iteration count can be expressed by Equation (3).

(3) $$r_{G}=s_{M}-(\frac{2\times s_{M}\times t}{2\times T}),$$

where the value of $s_{M}$ was inspired by the auditory characteristics of sand cats, so the original author set it to 2. t indicates the current iteration count, T indicates the maximum iteration count. Most importantly, it employs a parameter R to control the transition between the exploration and exploitation phases, which can be calculated using Equation (4).

(4) $$R=2\times r_{G}\times rand-r_{G},$$

where rand represents a random number between 0 and 1. Additionally, to realistically simulate the search process of sand cats, each sand cat is assigned a distinct search sensitivity, which can be calculated for each individual using Equation (5).

(5) $$r_{i}=r_{G}\times rand,$$

where $r_{i}$ indicates the search sensitivity of i-th sand cat, and use it for position updates during the algorithm’s exploration and development phases. $r_{G}$ is used to guide parameter R for controlling both the exploration and exploitation phases.

During the algorithm’s exploration phase, updates to new positions are influenced by both the current position and the optimal position. Additionally, different sand cats exhibit varying degrees of search sensitivity, which also guides their own position updates. In this phase, the mathematical model for updating a sand cat’s new position can be expressed as Equation (6).

(6) $$x_{i}^{t+1}=r_{i}\cdot (x_{best}^{t}+rand\cdot x_{i}^{t}),$$

where $x_{i}^{t}$ represents the position of the sand cat i during iteration t, $x_{i}^{t+1}$ represents the position of the sand cat i during iteration t+1, $x_{best}^{t}$ represents the position of the sand cat currently occupying the optimal location.

### 2.3. Attacking on the Prey (Exploitation)

After locating prey, the sand cat must attack to capture it. Since the sand cat detects prey through its ears, the author assumes its sensitivity range as a circle, allowing the sand cat’s movement direction to be controlled via a random angle on the circle. Based on this, the sand cat’s prey-attacking behavior can be modeled using Equation (7).

(7) $$x_{i}^{t+1}=x_{best}^{t}-r_{i}\cdot P\cdot cos(\theta ),$$

where P represents a random value for the distance between the optimal position and the current position, which can be calculated using Equation (8). θ is a random angle between 0 and 360 degrees.

(8) $$P=|rand\cdot x_{best}^{t}-x_{i}^{t}|,$$

By using Equation (7), sand cats can move along different circular paths within the search space. The SCSO selects a random angle for each sand cat, enabling them to approach hunting locations.

### 2.4. Exploration and Exploitation

The exploration and development of SCSO are adjusted via parameters $r_{G}$ and R. These two parameters enable SCSO to switch between the two phases. Since parameter R depends on $r_{G}$, when the values of parameter $r_{G}$ are uniformly distributed, the value of R can also be well balanced. Therefore, execution between the two phases can be adaptively adjusted according to the problem. From Equation (4), we can see that the range of R is [$-r_{G}$, $r_{G}$]. Since the value of $r_{G}$ decreases linearly from 2 to 0, to balance exploration and exploitation, the author sets the sand cat to exploit when R falls within the range [−1,1] and to explore otherwise. The specific mathematical model can be expressed by Equation (9).

(9) $$x_{i}^{t+1}=\left\{\begin{array}{c} x_{best}^{t}-r_{i}\cdot P\cdot \cos\left(\theta \right),\;if\left|R\right|\leq 1,\;exploitation\\ r_{i}\cdot \left(x_{best}^{t}+rand\cdot x_{i}^{t}\right),\;else,\;exploration \end{array}\right..$$

The pseudocode of the SCSO is outlined in Algorithm 1.
**Algorithm 1:** the pseudo-code of the SCSO1: Begin 2: Initialize: the relevant parameters and population initialization. 3: Calculate the fitness value for each individual and determine the optimal individual. 4:  While t <T do 5:    for i≤N do 6:      Randomly select an angle between 0 and 360 degrees. 7:      $\mathrm{if}\;\left|R\right|\leq 1$ 8:         Attacking on the prey (exploitation): 8:         Update the population by Equation (7) 9:      else: 10:         Searching the prey (exploration): 11:        Update the population by Equation (6) 12:      End If 16:      $\mathrm{Update}\;\mathrm{Parameters}\;r_{i}$, R$,\;r_{G}$ 19:    End for 20:   t=t+1 21:  End while 22:  return best solution 23: end

## 3. Proposed MISCSO

Although the original SCSO has demonstrated satisfactory performance, it often gets stuck in local optima when addressing the economic dispatch problem of microgrids that we need to solve. Therefore, to address its shortcomings, we propose three improvement strategies that comprehensively enhance the SCSO algorithm in three aspects: population initialization, exploration, and boundary control. The following sections will provide detailed introductions to these three strategies.

### 3.1. Distribution Optimization Initialization Based on Adaptive Diversity Guidance

**Figure 1:**
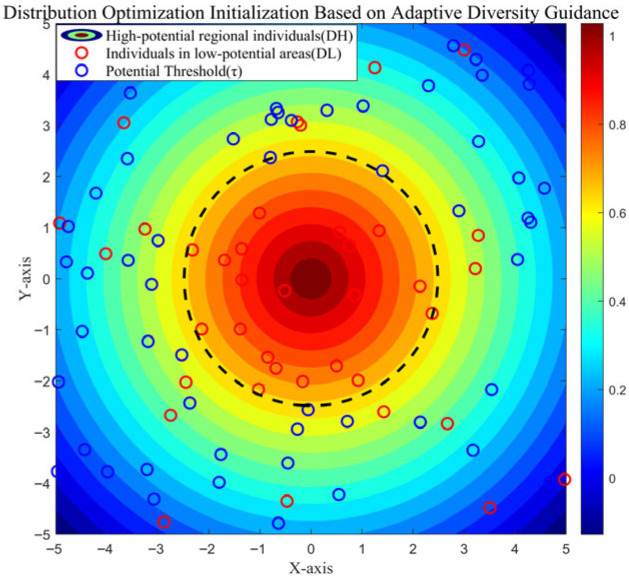
Distribution optimization initialization based on adaptive diversity guidance.

For a heuristic algorithm, the initial positions of individuals significantly impact its performance. Poor-quality initial solutions often prevent the algorithm from exploring effectively during early iterations, further slowing its convergence speed. During the algorithm’s initialization phase, obtaining a high-quality solution is our primary objective. To achieve this, we propose a distribution optimization initialization based on adaptive diversity guidance to secure favorable initial solutions during population initialization [15]. This strategy generates a portion of individuals in high-potential regions to ensure solution quality while simultaneously producing individuals in low-potential regions to enhance diversity. This approach effectively balances exploration and exploitation. The detailed mathematical model can be expressed by Equation (10).

(10) $$D=D_{H}\cup D_{L}$$

where $D_{H}$ denotes the high-potential individual group, while $D_{L}$ denotes the low-potential individual group. $D_{H}$ and $D_{L}$ are calculated using Equation (11) and Equation (12), respectively.

(11) $$D_{H}=\left\{x\in \Omega |\tilde{S}\left(x\right)\geq \tau \right\},\;|D_{L}|=N_{H}$$

(12) $$D_{L}=\left\{x\in \Omega |\tilde{S}\left(x\right)<\tau \right\},\;|D_{L}|=N_{L}$$

where $N_{H}=\lfloor \gamma N\rfloor$ and $N_{L}=N-N_{H}$ represent the number of high-potential individuals and low-potential individuals, respectively. γ is the adaptive weight, which can be expressed as Equation (13).

(13) $$\gamma =\gamma_{min}+\frac{\gamma_{max}-\gamma_{min}}{1+e^{-k(D_{cur}-D_{target})}}$$

where $D_{cur}$ represents the current population diversity, $D_{target}$ represents the reference population diversity, and k denotes the adjustment parameter. τ represents the high-potential threshold, which can be calculated using Equation (14).

(14) $$\tau =Q_{q}(\tilde{S}(x)),q\in [0.7,0.9]$$

where $\tilde{S}(x)$ denotes the potential function, which can be computed using Equation (15). Ω denotes the set of all positions. $Q_{q}(\cdot )$ denotes the quantile function, which determines the corresponding quantile value q from the output of the latent function $\tilde{S}(x)$.

(15) $$\tilde{S}(x)=\frac{S(x)-S_{min}}{S_{max}-S_{min}},\tilde{S}(x)\in [0,1]$$

where S(x) represents the fitness value at position x, while $S_{max}$ and $S_{min}$ denote the maximum and minimum fitness values among all values, respectively. A schematic of the distribution optimization initialization based on adaptive diversity guidance in Figure 1.

### 3.2. An Elite-Centered Global Random Movement Strategy

During the exploration phase of an algorithm, if position updates rely solely on the optimal value, the likelihood of the algorithm becoming trapped in local optima significantly increases. To address this shortcoming, we propose an Elite-Centered Global Random Movement Strategy to enhance the exploration component of SCSO. In this proposed strategy, position updates depend not only on the optimal solution but also on suboptimal solutions, centroids, and other factors. The specific mathematical model can be expressed as Equation (16).

(16) $$x_{i,j}^{t+1}={Elite}_{K1,j}+{RA}_{i,j}\times (r_{1}*\left(x_{best}^{t}-x_{i,j}^{t}\right)+\left(1-r_{1}\right)\times \left(x_{cent}^{t}-x_{i,j}^{t}\right)),$$

where Elite denotes the elite pool, which contains the optimal solution, the second-best solution, the third-best solution, and the average of the top half of the individuals. ${RA}_{i,j}$ generates random numbers with a mean of 0 and a standard deviation of 1, enhancing the algorithm’s ability to escape local optima. $r_{1}$ represents a random number between 0 and 1, serving as the coefficient for the difference between the optimal position and the current position. $x_{cent}^{t}$ indicates the position of the center point of the target, which can be calculated using Equation (17). K1 represents a random integer between 1 and 4, used to randomly select individuals from the elite pool.

(17) $$x_{cent}^{t}=\frac{1}{N}\sum_{i=1}^{N}x_{i,j}^{t},$$

**Figure 2:**
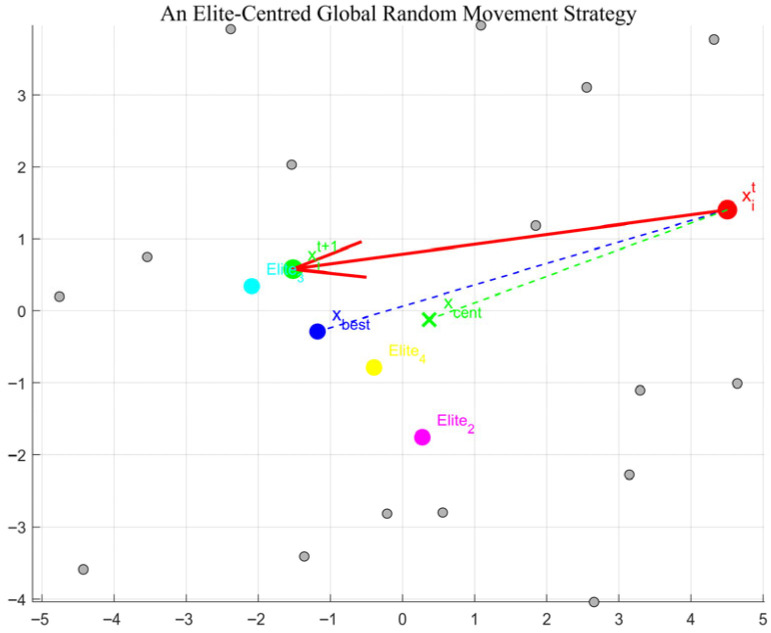
A schematic of the elite-centered global random movement strategy.

A schematic of the elite-centered global random movement strategy in Figure 2.

### 3.3. Adaptive Elastic Boundary Mapping Method

In optimization algorithms, boundary handling is one of the core issues determining algorithm performance. When individuals exceed the predefined search/feasible domain boundaries during iteration or movement, traditional boundary handling methods often employ truncation or random reset techniques, which frequently exhibit limitations: the former causes individuals to cluster near boundaries, losing diversity; the latter disrupts the continuity of individual movement, reducing algorithm stability. To address this issue, we treat boundaries as “elastic media” rather than rigid barriers. When an agent crosses a boundary, its subsequent position and velocity are adaptively calculated based on the distance of the crossing. This approach ensures the agent remains within the feasible domain while preserving motion continuity and the algorithm’s exploration capability. This can be modeled as expressed in Equation (18).

(18) $$x_{i}^{t}=\left\{\begin{array}{cc} lb+\alpha_{i}\times \left(lb-x_{i}^{t}\right),\; & x_{i}^{t}<lb\\ ub-\alpha_{i}\times \left(x_{i}^{t}-ub\right),\; & x_{i}^{t}>ub \end{array}\right.$$

where $\alpha_{i}$ denotes the adaptive elasticity coefficient. It can be calculated using Equation (19).

(19) $$\alpha_{i}=\left(\alpha_{min}+\left(\alpha_{max}-\alpha_{min}\right)\times \frac{t}{T}\right)\times (1+\lambda \times \delta_{i})$$

where $\alpha_{max}$ and $\alpha_{min}$ represent the maximum and minimum values of the elastic modulus. λ represents the weighting coefficient for the degree of boundary violation. 111 represents the boundary crossing coefficient, which can be calculated using Equation (20).

(20) $$\delta_{i}=\left\{\begin{array}{cc} \frac{lb-x_{i}^{t}}{ub-lb},\; & x_{i}^{t}<lb\\ \frac{x_{i}^{t}-ub}{ub-lb},\; & x_{i}^{t}>ub \end{array}\right.$$

**Figure 3:**
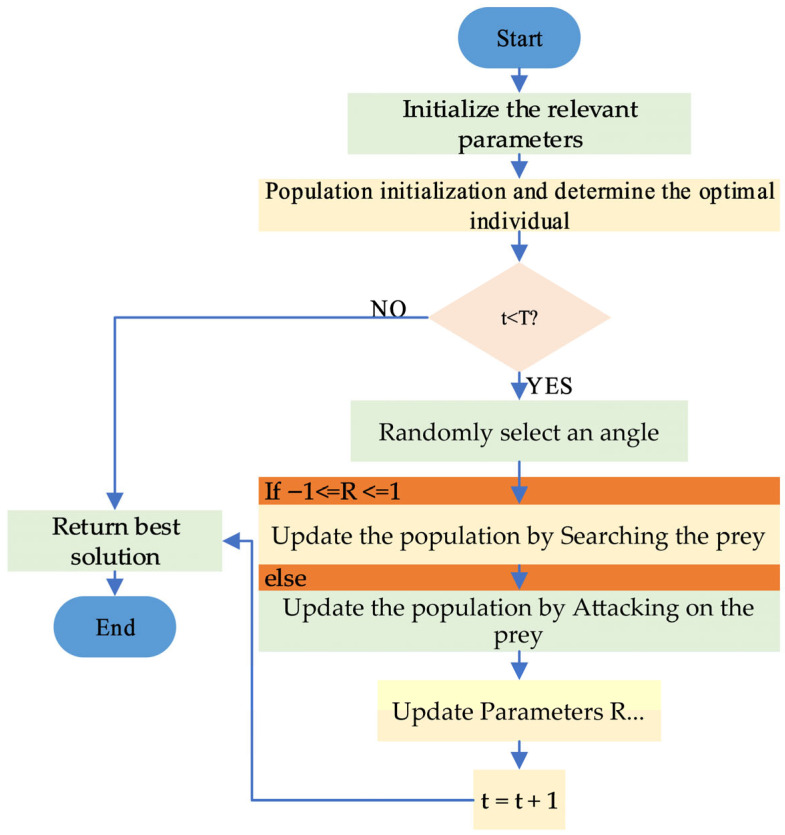
Flowchart of MISCSO.

Figure 3 depicts the flowchart of the MISCSO.

## 4. Experimental Results and Detailed Analyses on CEC2017

In this section, we carry out experiments on the IEEE CEC2017 test suite to assess the performance of MISCSO. First, we provide a brief overview of the test suite, the comparison algorithms, and their parameter configurations. Next, we verify the performance of MISCSO through comparisons with other optimization algorithms. Finally, to identify whether there are significant differences between MISCSO and the other algorithms, we utilize two statistical testing methods for analysis. The specific details are elaborated as follows.

### 4.1. Benchmark Test Functions

The IEEE CEC2017 test suite is a standard benchmark collection in the field of evolutionary computation for evaluating the performance of optimization algorithms, possessing high authority and broad applicability [16]. The CEC2017 test suite comprises 30 test functions, primarily categorized into unimodal functions, multimodal functions, hybrid functions, and composite functions. The test set covers a broad spectrum of problem difficulty and characteristics, ranging from simple functions to highly complex nonlinear and nonconvex functions. Widely used in academic research, it presents a significant challenge to algorithms while enjoying high academic recognition. It provides a unified testing platform for optimization algorithm researchers worldwide, facilitating objective and fair comparisons of different algorithms’ strengths and weaknesses. Therefore, this paper selects IEEE CEC2017 as the benchmark for algorithm testing.

### 4.2. Competitor Algorithms and Parameter Setting

In this section, we evaluate the performance of the MISCSO by comparing it with 11 advanced algorithms to demonstrate its strong capabilities. The algorithms used for comparison include particle swarm optimization (PSO), dung beetle optimizer (DBO), dwarf mongoose optimization algorithm (DMO), information acquisition optimizer (IAO), moss growth optimization (MGO), parrot optimizer (PO), polar lights optimizer (PLO), black-winged kite algorithm (BKA), hybridization of constriction coefficient based on particle swarm optimization and gravitational search algorithm (CPSOGSA), hybrid parallel harris hawks optimization algorithm (HPHHO), and sand cat optimization algorithm (SCSO). For the sake of experimental fairness, all comparison algorithms were assigned a consistent population size of 50 and a maximum iteration number of 1000. To mitigate randomness in the algorithms, each was independently executed 30 times, and the results were averaged for analysis. Table 1 summarizes the parameter configurations of these algorithms for enhanced readability.

### 4.3. Compare Using CEC 2017 Test Functions

**Figure 4:**
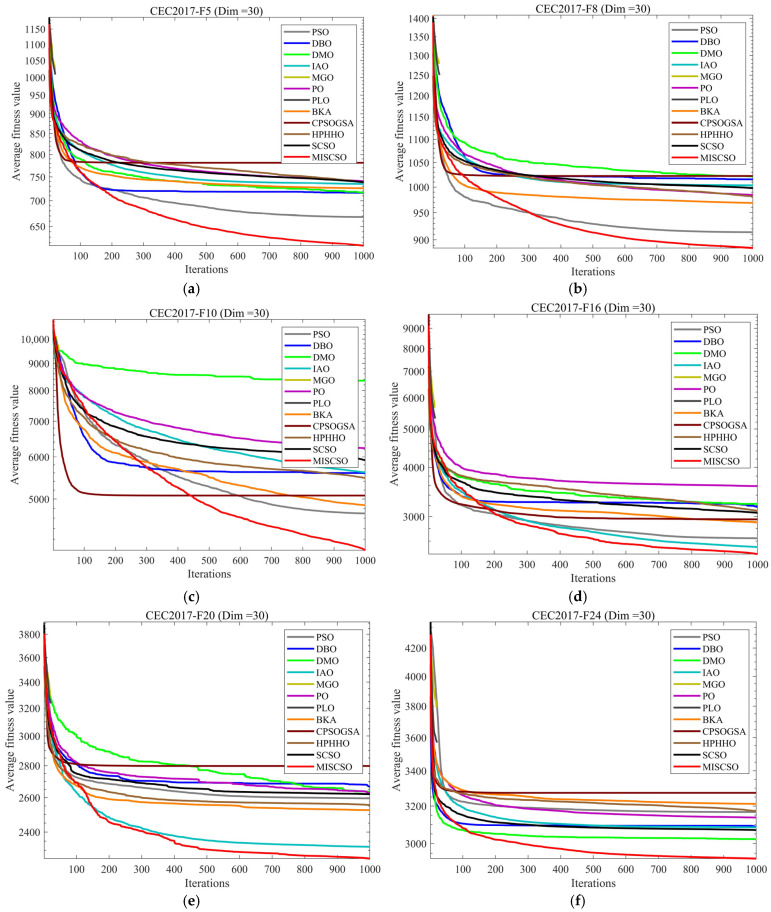

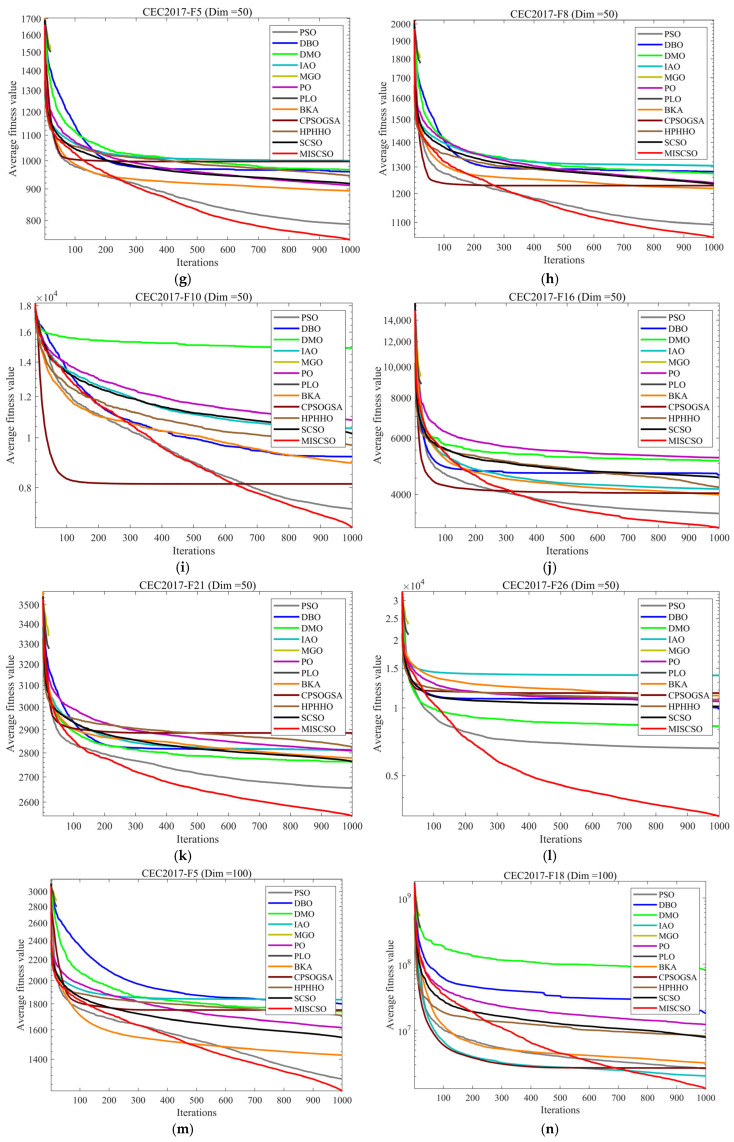

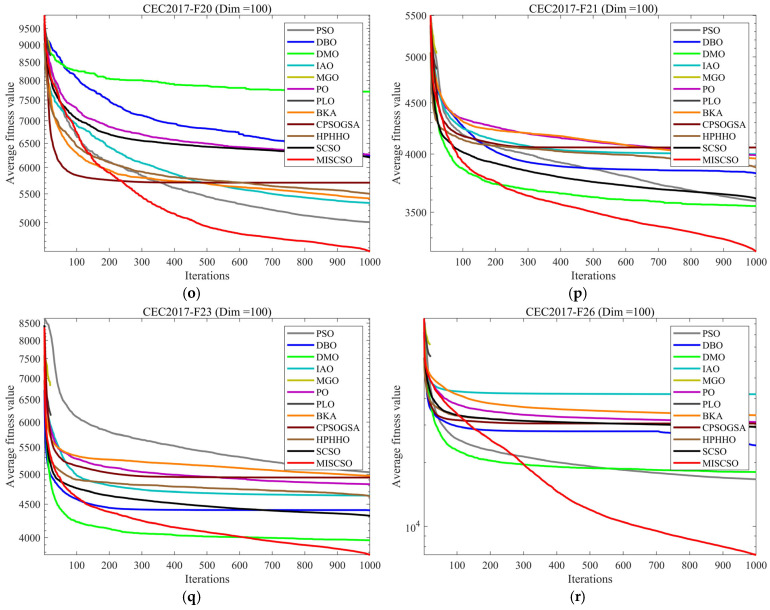
Comparison of convergence speed of different algorithms on CEC2017 test set. (**a**) CEC2017-F5 (Dim = 30), (**b**) CEC2017-F8 (Dim = 30), (**c**) CEC2017-F10 (Dim = 30), (**d**) CEC2017-F16 (Dim = 30), (**e**) CEC2017-F20 (Dim = 30), (**f**) CEC2017-F24 (Dim = 30), (**g**) CEC2017-F5 (Dim = 50), (**h**) CEC2017-F8 (Dim = 50), (**i**) CEC2017-F10 (Dim = 50), (**j**) CEC2017-F16 (Dim = 50), (**k**) CEC2017-F21 (Dim = 50), (**l**) CEC2017-F26 (Dim = 50), (**m**) CEC2017-F5 (Dim = 100), (**n**) CEC2017-F18 (Dim = 100), (**o**) CEC2017-F20 (Dim = 100), (**p**) CEC2017-F21 (Dim = 100), (**q**) CEC2017-F23 (Dim = 100), (**r**) CEC2017-F26 (Dim = 100).

**Figure 5:**
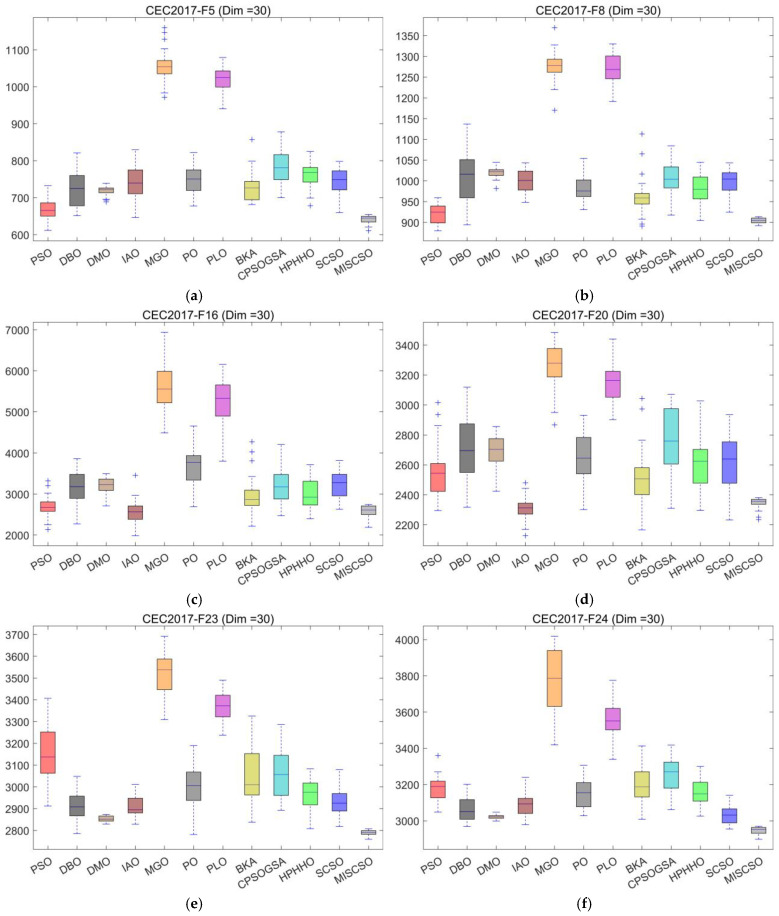

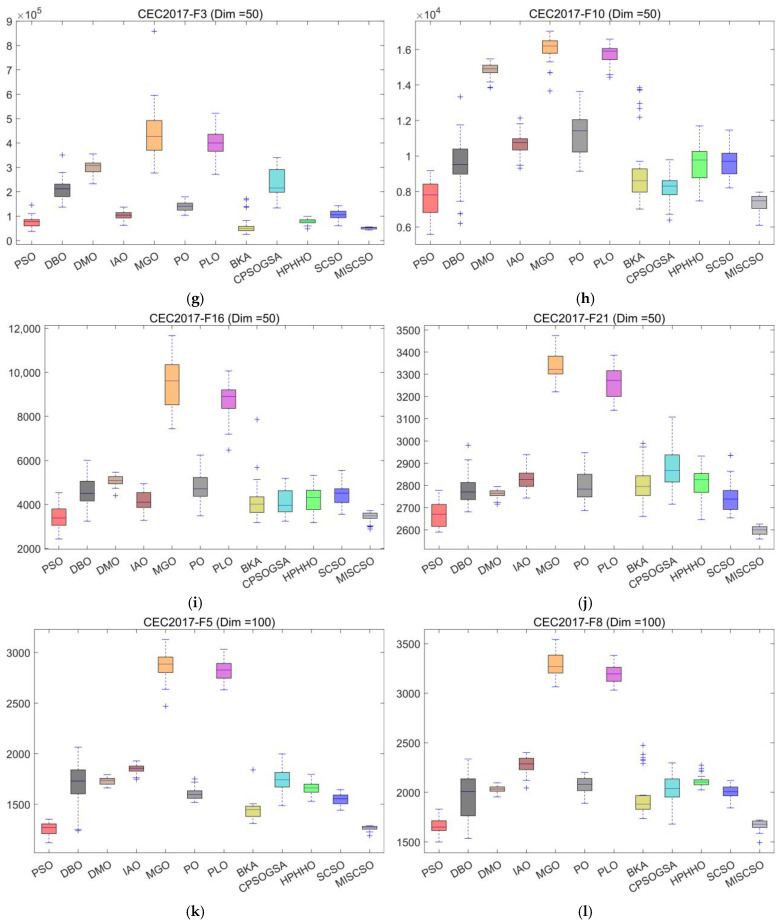

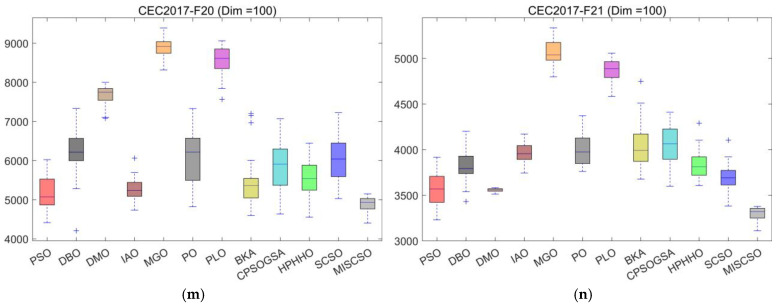
Boxplot analysis for different algorithms on the CEC2017 test set. (**a**) CEC2017-F5 (Dim = 30), (**b**) CEC2017-F8 (Dim = 30), (**c**) CEC2017-F16 (Dim = 30), (**d**) CEC2017-F20 (Dim = 30), (**e**) CEC2017-F23 (Dim = 30), (**f**) CEC2017-F24 (Dim = 30), (**g**) CEC2017-F3 (Dim = 50), (**h**) CEC2017-F10 (Dim = 50), (**i**) CEC2017-F16 (Dim = 50), (**j**) CEC2017-F21 (Dim = 50), (**k**) CEC2017-F5 (Dim = 100), (**l**) CEC2017-F8 (Dim = 100), (**m**) CEC2017-F20 (Dim = 100), (**n**) CEC2017-F21 (Dim = 100).

To showcase the performance advantages of MISCSO, this section implements experimental analysis using the CEC2017 test set, where MISCSO is compared with 11 benchmark algorithms. Figure 4 depicts the average convergence curves of all 12 algorithms after 30 independent runs. Table 2, Table 3 and Table 4 report the three-dimensional performance metrics of the 12 algorithms on the CEC2017 test set. Here, “Mean” indicates the average of the 30 independent runs, and “Std” reflects the standard deviation of each algorithm’s results across the 30 independent runs. To visually exhibit the results and underscore MISCSO’s performance superiority, Figure 5 presents box plots derived from the 30 independent runs of each algorithm.

As shown in Figure 4, the MISCSO algorithm generally exhibits a faster rate of decline in the average fitness value during the convergence process of various functions. It converges more rapidly toward optimal solutions, and its final stable average fitness value is significantly lower than that of most benchmark algorithms. Its distinctive feature lies in its robust search and convergence capabilities across monomodal, multimodal, and composite functions. It rapidly reduces fitness values during early iterations while maintaining stable performance at lower levels thereafter, demonstrating exceptional efficiency and robustness in solving complex optimization problems. This provides a clear advantage over other algorithms. For functions with 50 dimensions, MISCSO’s average fitness value rapidly declines as iterations increase, converging faster than most benchmark algorithms and stabilizing at a lower level. Even when dimensionality increases to 100, MISCSO maintains a significant advantage. Its convergence curve exhibits a steep initial decline and sustains a relatively low average fitness value throughout the process, demonstrating robust search capability and resilience even in high-dimensional complex problem spaces.

The data in the table shows that for most test functions, MISCSO’s “mean” value is significantly lower than that of many other algorithms. A lower “mean” value indicates that MISCSO achieves better optimization results on average. Additionally, MISCSO exhibits relatively low “std” values, indicating greater stability and reduced performance variability across multiple independent runs. This confirms its effectiveness in tackling complex optimization problems.

Figure 5 illustrates the performance distribution of the MISCSO algorithm alongside 11 benchmark algorithms (PSO, DBO, DMO, IAO, MGO, PO, PLO, BKA, CPOSGSA, HPHHO, SCSO) across multiple functions and varying dimensions within the CEC2017 test set. At 30 dimensions, MISCSO’s box plot occupies a lower position compared to most benchmark algorithms. At 50 dimensions, it maintains a lower median and narrower box range. At 100 dimensions, MISCSO’s box plot remains at a low level with minimal fluctuation. This demonstrates MISCSO’s strong scalability, enabling it to effectively handle high-dimensional complex optimization problems. In summary, box plot analysis demonstrates that across different functions and dimensions in the CEC2017 test set, the MISCSO algorithm exhibits significant advantages over other benchmark algorithms in both solution accuracy and stability, validating its effectiveness in solving complex optimization problems.

### 4.4. Statistical Analysis

To ascertain if there are notable disparities between the proposed algorithm and other algorithms, statistical analyses are carried out on each algorithm in this section. Firstly, the Wilcoxon signed-rank test is utilized to assess the 12 algorithms and compare their significant differences. Then, the Friedman test of median ranks is adopted to evaluate the ranking performance of each algorithm. The detailed results are presented as follows:

#### 4.4.1. Wilcoxon Rank Sum Test

In this section, we conducted the Wilcoxon rank sum test on 12 algorithms [27]. The Wilcoxon Rank Sum Test is a nonparametric statistical test used to determine whether there is a significant difference between two independent samples without requiring the data to follow a specific distribution. It offers advantages such as broad applicability and relatively lenient data requirements. In this experiment, we set the significance level to 0.05. If p<0.05, we reject the null hypothesis, indicating a significant difference; otherwise, we accept the null hypothesis, suggesting no significant difference.

Table 5, Table 6 and Table 7 present experimental results across three dimensions for 11 comparison algorithms on the CEC2017 test set. The data reveal that MISCSO exhibits significant differences from the comparison algorithms on most test functions.

#### 4.4.2. Friedman Mean Rank Test

In this subsection, we applied the Friedman mean rank test to 12 algorithms [28]. The Friedman mean rank test is another nonparametric statistical method primarily used to assess whether significant differences exist among multiple related samples. It is frequently applied to evaluate performance variations among different algorithms. This test is agnostic to the distribution shape of the data and does not require assumptions such as normal distribution, making it widely applicable. Therefore, we used it to test the performance of various comparison algorithms and rank them. Table 8 displays the average rankings of the 12 algorithms across 30 test functions in three dimensions. Among these, M.R represents the average ranking of each algorithm across 30 test functions, while T.R denotes the final ranking of each algorithm across 30 test functions.

The experimental results show that the MISCSO algorithm achieved the top ranking in all three scenarios with dimensions of 10, 30, and 50. Additionally, its average rankings were 2.33, 2.33, and 2.40, respectively, the lowest among all algorithms. In contrast, the SCSO algorithm achieved final rankings of 9th, 9th, and 8th across the three dimensions. This indicates that MISCSO represents a significant improvement over SCSO, clearly demonstrating its substantial advantage in the CEC2017 test set. MISCSO outperformed all other comparison algorithms across different dimensionality settings.

## 5. MISCSO for Economic Scheduling of Microgrids

In the current era where the digital economy deeply permeates the energy sector, microgrid economic dispatch is transitioning from traditional experience-driven models to data-driven intelligent approaches. The core driving force behind this transformation lies in the deep integration of digital technologies and innovative management. Leveraging big data, the Internet of Things, artificial intelligence, and other technologies as connecting links, the digital economy enables real-time collection and dynamic analysis of massive datasets—including distributed energy, energy storage equipment, and load demand. This enables precise load forecasting and multi-energy complementary optimization algorithms for microgrid economic dispatch. It not only reduces operational costs through peak-valley electricity arbitrage but also enhances clean energy consumption rates via source-load coordination, elevating economic dispatch from “rough balancing” to “precise regulation.” Simultaneously, innovative management models provide institutional safeguards for this technological empowerment. By establishing cross-entity data-sharing mechanisms, information barriers across energy production, transmission, and consumption are dismantled. Agile organizational structures bring dispatch decisions closer to dynamic user-side demands. Market-based incentive mechanisms encourage user participation in demand response, creating a dual-engine drive of “technological optimization + management innovation.” It can be said that the digital economy has infused the technical core into microgrid economic dispatch, while innovative management has built an efficient institutional framework for its operation. The synergistic efforts of these three elements not only reshape the economic logic of energy systems but also serve as a key driver for the deep integration of the energy revolution and the digital revolution.

In this section, to address the economic dispatch optimization problem for microgrids, we first establish a mathematical model of the micro-power source system. This model incorporates photovoltaic power generation, wind power generation, energy storage devices, and diesel generators. Subsequently, an objective function is formulated based on economic and environmental costs to guide the optimization process. The specific implementation steps are as follows:

### 5.1. Micro-Power Mathematical Model

In this subsection, we construct the mathematical model for the micro-power source system using models of the photovoltaic power generation system, wind power generation system, energy storage device, and diesel generator. The specific details are as follows:

#### 5.1.1. Photovoltaic Power Generation Model

Photovoltaic power generation is a system that utilizes the photovoltaic effect to convert solar energy into electrical energy. The power output of the generating device is primarily influenced by factors such as temperature and illumination. Therefore, the photovoltaic power generation system model can be calculated using Equation (21).

(21) $$P_{PV}=P_{STC}\frac{G_{C}}{G_{STC}}[1+k(T_{C}-T_{STC})],$$

where $P_{STC}$ denotes the rated power under standard conditions; $G_{C}$ and $G_{STC}$ represent the actual illuminance and illuminance under standard conditions, respectively; $T_{C}$ and $T_{STC}$ denote the operating temperature and standard temperature, respectively. k denotes the power temperature coefficient, which characterizes how the output power of photovoltaic cells varies with temperature. Its value is −0.4%/°C.

#### 5.1.2. Wind Power Generation System Model

Wind power generation systems primarily consist of wind turbines, whose output power is influenced by factors such as wind speed, wind force, and turbine location. Therefore, the wind power generation system model can be expressed using Equation (22).

(22) $$P_{WT}=\left\{\begin{array}{c} 0,\;\;\;u⩽u_{i},\\ P_{WT,rate}\frac{u-u_{i}}{u_{r}-u_{i}},\;\;\;\;\;\;u_{i}⩽u⩽u_{r},\\ P_{WT,rate},\;\;\;u_{r}⩽u⩽u_{o},\\ 0,\;\;\;u⩾u_{o} \end{array}\right.$$

where $P_{WT,rate}$ denotes the fan’s rated output power; $u_{i}$, u, and $u_{r}$ represent the cut-in wind speed, cut-out wind speed, and rated wind speed, respectively.

#### 5.1.3. Energy Storage Device Model

Energy storage devices can effectively ensure the stable operation of microgrids and also achieve peak shaving and valley filling functions. The electrical energy stored in the energy storage system is related to the state of charge (SOC) of the batteries. Its charging and discharging model at time t is shown in Equation (23).

(23) $$\left\{\begin{array}{c} f_{SOC}\left(t\right)=f_{SOC}\left(t-1\right)+P_{k}\left(t\right)\eta_{c},\;\;\;P_{k}(t)>0,\\ f_{SOC}\left(t\right)=f_{SOC}\left(t-1\right)+P_{k}\left(t\right)\eta_{d},\;\;\;P_{k}(t)⩽0, \end{array}\right.$$

where $f_{SOC}\left(t\right)$ and $f_{SOC}\left(t-1\right)$ represent the remaining battery capacity at times t and t−1, respectively; $P_{k}\left(t\right)$ indicates the charging/discharging power of the energy storage device; $P_{k}\left(t\right)>0$ denotes charging status, while $P_{k}\left(t\right)\leq 0$ indicates discharging status; $\eta_{c}$ and $\eta_{d}$ represent charging and discharging efficiency, respectively.

#### 5.1.4. Diesel Generator Model

The power generation cost of diesel generators is expressed by a quadratic function as shown in Equation (24).

(24) $$C_{DE}=a+bP_{DE}+c{P_{DE}}^{2},$$

where $C_{DE}$ represents fuel costs, $P_{DE}$ represents the power output of diesel generators; a, b, and c represent fuel cost coefficients, respectively.

### 5.2. Objective Function

The objective function aims to minimize economic costs while maximizing environmental benefits. Economic costs primarily encompass operational expenses, fuel costs, and electricity exchange costs with the grid. Environmental costs include pollution control expenses for micro-power sources (CO_2_, NO_2_, SO_2_). The objective function to minimize economic and environmental control costs can be expressed as Equation (25).

(25) $$F=min(\varphi F_{1}+\mu F_{2}),$$

where F represents the total operating cost; $F_{1}$ and $F_{2}$ denote economic cost and environmental remediation expenses, respectively; φ and μ are the coefficients for economic cost and environmental remediation expenses, both set at 0.5.

#### 5.2.1. Economic Cost

The specific economic costs can be calculated based on the fuel coefficients and operational management expenses of each micro power source, and can be expressed as shown in Equation (26).

(26) $$minF_{1}(x)=\sum_{t=1}^{T}\left[\sum_{i=1}^{N}(C_{i,f}P_{i,t}+C_{i,m}P_{i,t})+C_{GRID,t}P_{GRID,t}\right],$$

where T denotes a scheduling cycle, N indicates the type of distributed power source; $C_{i,f}$ and $C_{i,m}$ represent the fuel coefficient and operational management fee for each micro-power source, respectively; $P_{i,t}$ is the output power of the i-th micro-power source; $C_{GRID,t}$ is the electricity price at time t; $P_{GRID,t}$ is the power exchanged with the grid at time t.

#### 5.2.2. Environmental Costs

Environmental costs are primarily determined by the expenses incurred in treating various pollutants, which can be specifically expressed as Equation (27).

(27) $$minF_{2}\left(x\right)=\sum_{t=1}^{T}\left[\sum_{i=1}^{N}\left(\sum_{h=1}^{M}\beta_{i,h}\alpha_{i,h}P_{i,t}\right)\right],$$

where M represents the pollutant type; $\beta_{i,h}$ denotes the emission factor for pollutant h emitted by distributed power source i; $\alpha_{i,h}$ indicates the abatement cost for pollutant h emitted by distributed power source i.

### 5.3. Constraints

In this subsection, we employ power balance, the interaction power between the microgrid and the main grid, and the battery as constraints in solving the optimization problem. These constraints are specifically expressed as Equations (28)–(31).

(28) $$P_{load}^{\left(t\right)}=\sum_{i=1}^{N}P_{Gi}^{\left(t\right)}+P_{GRID}^{\left(t\right)},$$

(29) $$P_{GRID}^{min}⩽P_{GRID}^{\iota }⩽P_{GRID}^{max}(t),$$

(30) $$P_{i}^{min}(t)⩽P_{i}^{t}(t)⩽P_{i}^{max}(t),$$

(31) $$f_{SOC,min}⩽f_{SOC,t}⩽f_{SOC,max},$$

where Equation (28) represents the power balance constraint, Equation (29) denotes the power interaction constraint between the microgrid and the main grid, Equation (30) indicates the output constraints for each micro-source, and Equation (31) specifies the constraints for the battery. $P_{load}^{\left(t\right)}$ represents the total power load at time t; $P_{Gi}^{\left(t\right)}$ represents the output power of each micro-power source at time t; $P_{GRID}^{\left(t\right)}$ represents the power exchanged with the grid at time t. $P_{GRID}^{min}$ and $P_{GRID}^{max}$ represent the minimum and maximum power levels for mainnet interactions, respectively. $P_{i}^{min}(t)$ and $P_{i}^{max}(t)$ represent the minimum and maximum output power values of each micro power source at time t, respectively. $f_{SOC,min}$ and $f_{SOC,max}$ represent the upper and lower limits of the battery’s state of charge, respectively.

### 5.4. Simulation Parameter Settings and Experimental Analysis

#### 5.4.1. Simulation Parameters for the Case Study

This study primarily investigates grid-connected microgrids, utilizing MATLAB 2023a as the simulation platform. GRID represents the power grid. To ensure a fair comparison among algorithms, the population size is set to 50, with an interaction power of 200 kW between the microgrid and the main grid. The operational parameters of each micro-power source and the pollutant treatment costs are presented in Table 9 and Table 10, respectively.

During microgrid grid-connected operation, grid electricity prices are based on real-time pricing, disregarding electricity sales. The real-time electricity purchase and sale unit prices for each time period are shown in Table 11.

#### 5.4.2. Analysis of Experimental Results

**Figure 6:**
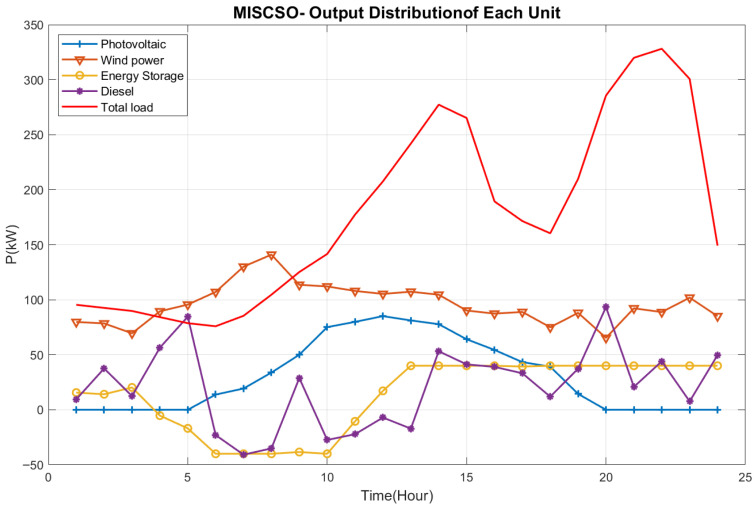
Output Power of Each Power Supply After MISCSO.

**Figure 7:**
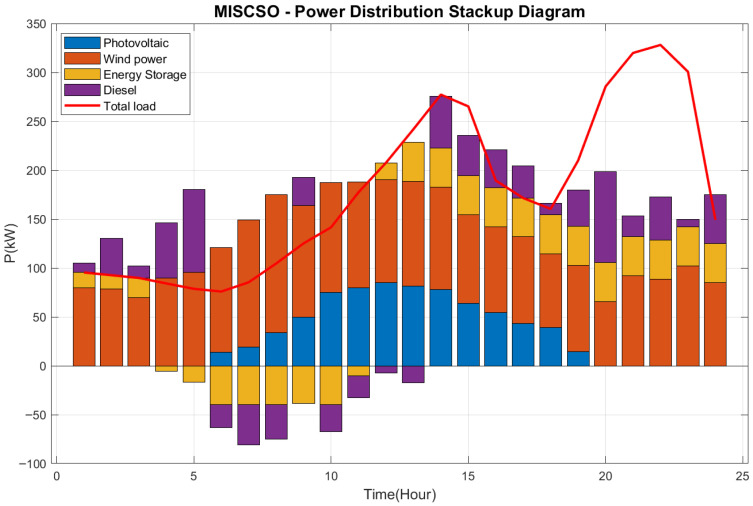
Power Distribution Stackup Diagram.

This study examines a typical day in a specific region of Guangdong. To ensure experimental fairness and eliminate randomness, each algorithm was independently run 30 times. Experimental statistics include the maximum value (Max), minimum value (Min), mean (Ave), standard deviation (Std), and ranking (Rank), as shown in Table 12. The output of each distributed power source is illustrated in Figure 6. Figure 7 shows the power allocation stacking diagram planned by the MISCSO algorithm.

The data in the table demonstrates that the MISCSO algorithm exhibits significant advantages across all key metrics. Regarding minimum cost (Min), MISCSO achieved 4986.87, the lowest value among all compared algorithms. For instance, the minimum cost of the PSO algorithm was 5026.37, while that of the DBO algorithm was 6153.18, both exceeding MISCSO’s result. This fully demonstrates MISCSO’s outstanding capability in locating the lowest-cost solution. For mean cost (Mean), MISCSO’s average of 5495.67 remains the lowest among all algorithms. Other algorithms, such as PSO with an average cost of 6571.94 and DBO at 6765.53, exhibit significantly higher mean costs than MISCSO, indicating its outstanding overall cost performance after multiple iterations. In terms of stability (Std), MISCSO exhibits the smallest standard deviation at 242.91 among all algorithms. In contrast, algorithms like PSO (standard deviation 679.16) and DBO (537.96) demonstrate significantly greater cost fluctuations than MISCSO. This indicates that MISCSO delivers more stable costs per run and stands out for its consistency in results. Regarding the overall ranking (Rank), MISCSO achieved the highest position at 1st place among all algorithms. This directly demonstrates that under the objective of “cost minimization,” MISCSO delivers the most optimal comprehensive performance compared to all other algorithms. When specifically compared against typical algorithms like PSO, DBO, and MGO, MISCSO exhibits lower minimum and average costs than these algorithms while maintaining superior stability, resulting in the best overall ranking. In summary, across the four core dimensions of minimum cost, average cost, stability, and overall ranking, the MISCSO algorithm demonstrates significant advantages over all comparison algorithms: it exhibits the strongest ability to find optimal solutions, achieves the lowest overall cost, and delivers the most stable results. It is the algorithm with the best overall performance in this comparison. Based on CPU execution time, MISCSO ran for 201.08 s, ranking third among the 12 algorithms. Its CPU time exceeded that of SCSO by less than two seconds. Given the performance improvement achieved, this time overhead is acceptable.

As shown in Figure 6, photovoltaic power generation exhibits distinct peaks and troughs in output between 0 and 25 h. For instance, it reaches a higher output (exceeding 300 kW) around 20 h, while output drops significantly during certain periods (such as near 15 h), even turning negative. This may be related to variations in sunlight conditions, highlighting the significant influence of weather and time on photovoltaic power generation. Wind power: Overall output is relatively dispersed and fluctuates, with values generally ranging from −50 kW to 100 kW. This reflects the instability of wind power output due to varying wind conditions. Energy storage: Output fluctuates within a certain range, enabling energy storage or release during different periods to serve a regulatory function. For instance, output may be positive (releasing energy) during certain periods and negative (storing energy) during others. Diesel Generation: Output remains relatively stable, primarily fluctuating between 0 and 100 kW. As a conventional energy source, it provides a stable power supplement when renewable generation is insufficient. In terms of coordinating output from various units, the MISCSO algorithm enables optimized scheduling of energy units with differing characteristics, including photovoltaic power generation, wind power, energy storage, and diesel generation. When photovoltaic and wind power outputs fluctuate unpredictably, the system relies on energy storage regulation and diesel generation supplementation to ensure overall power supply effectively adapts to total load demands. This demonstrates the algorithm’s effectiveness in optimizing multi-energy system configurations, thereby enhancing energy utilization efficiency and power system stability.

Figure 7 illustrates the power output distribution of different energy units (photovoltaic power generation, wind power generation, energy storage, diesel power generation) over 0–25 h and their relationship with total load. The MISCSO algorithm enables efficient optimization of these energy units with distinct characteristics—photovoltaic power generation, wind power generation, energy storage, and diesel power generation. By rationally allocating the output of each energy source, the algorithm ensures that the overall power supply can effectively meet the total load demand. This is achieved through the regulation of energy storage and the supplementation of diesel power generation, even when intermittent energy sources like solar and wind exhibit unstable output. This demonstrates the algorithm’s effectiveness in optimizing the scheduling of multi-energy systems, thereby enhancing energy utilization efficiency and the stability of the power system.

Finally, Table 13 summarizes the full forms of abbreviations frequently used in this paper for the reader’s reference. Table 14 summarizes the symbols used in this paper.

## 6. Conclusions

In this paper, we propose a multi-strategy improved SCSO (MISCSO) to address the economic scheduling problem of microgrids. First, to enhance the quality of the initial population, a distribution-optimized initialization method based on adaptive diversity guidance is developed. This approach improves the algorithm’s performance by generating individuals in high-potential regions to ensure solution quality, while simultaneously producing individuals in low-potential regions to maintain population diversity. Subsequently, an elite-centered global random movement strategy is introduced to balance elite guidance and global exploration, thereby enhancing both the convergence speed and optimization accuracy of the algorithm. In addition, an adaptive elastic boundary mapping mechanism is proposed to effectively handle boundary violations, ensuring a balance between constraint satisfaction and global search capability. To evaluate the performance of the proposed MISCSO, we compared it with 11 advanced algorithms on the IEEE CEC2017 benchmark set, and conducted statistical analyses to assess performance differences. Experimental results demonstrated that MISCSO exhibits superior optimization ability and robustness. Finally, its practical applicability is verified by applying it to the economic scheduling of microgrids, where it achieves excellent optimization results.

## Figures and Tables

**Table 1:** Parameter settings of the comparison algorithms.

Algorithms	Parameter Name	Parameter Value	Reference
PSO	Vmax , wMax , wMin , c1 , c2	6, 0.9, 0.6, 2, 2	[17]
DBO	P_percent	0.2	[18]
DMO	peep	2	[19]
IAO	alpha	0.5	[20]
MGO	Rec, w, d1	1, 2, 0.2	[21]
PO	Beta	1.5	[22]
PLO	Beta	1.5	[23]
BKA	p	0.9	[24]
CPSOGSA	phi1	2.05	[25]
HPHHO	Rabbit_Energy	1 × 10^20^	[26]
SCSO	S	2	[12]

**Table 2:** Results of various algorithms tested on the CEC 2017 benchmark (dim = 30).

ID	Metric	PSO	DBO	DMO	IAO	MGO	PO	PLO	BKA	CPSOGSA	HPHHO	SCSO	MISCSO
F1	mean	2.2091 × 10^5^	1.8798 × 10^7^	1.1622 × 10^6^	2.2219 × 10^10^	8.3689 × 10^10^	5.1717 × 10^8^	7.6459 × 10^10^	7.8964 × 10^9^	2.8746 × 10^3^	9.9424 × 10^8^	4.2861 × 10^9^	8.9823 × 10^5^
	std	1.2262 × 10^5^	2.2615 × 10^7^	1.4200 × 10^6^	5.4481 × 10^9^	1.3990 × 10^10^	5.2480 × 10^8^	1.6098 × 10^10^	1.2665 × 10^10^	3.7008 × 10^3^	5.7386 × 10^8^	2.5945 × 10^9^	2.0484 × 10^5^
F2	mean	5.2899 × 10^9^	4.2621 × 10^28^	4.8542 × 10^31^	4.9180 × 10^29^	3.0023 × 10^46^	1.6438 × 10^31^	8.8433 × 10^44^	5.0686 × 10^39^	7.8063 × 10^27^	1.0000 × 10^20^	1.2044 × 10^33^	6.6077 × 10^13^
	std	1.2806 × 10^10^	1.6127 × 10^29^	1.6032 × 10^32^	1.3743 × 10^30^	9.9573 × 10^46^	7.1850 × 10^31^	3.1955 × 10^45^	2.7745 × 10^40^	4.0246 × 10^28^	0.0000 × 10^0^	4.8686 × 10^33^	5.2354 × 10^13^
F3	mean	5.6855 × 10^3^	6.7094 × 10^4^	1.1122 × 10^5^	3.7433 × 10^4^	2.2896 × 10^5^	4.1243 × 10^4^	1.9017 × 10^5^	1.1762 × 10^4^	6.5738 × 10^4^	3.3279 × 10^4^	4.6717 × 10^4^	8.6403 × 10^3^
	std	2.9498 × 10^3^	1.3185 × 10^4^	1.6079 × 10^4^	1.1193 × 10^4^	4.9962 × 10^4^	7.5511 × 10^3^	3.2697 × 10^4^	8.0095 × 10^3^	2.2409 × 10^4^	9.2237 × 10^3^	1.1329 × 10^4^	1.1697 × 10^3^
F4	mean	4.7371 × 10^2^	5.8079 × 10^2^	5.1558 × 10^2^	3.8787 × 10^3^	2.3917 × 10^4^	5.7897 × 10^2^	1.8050 × 10^4^	1.2642 × 10^3^	5.2289 × 10^2^	6.0051 × 10^2^	8.7244 × 10^2^	4.8154 × 10^2^
	std	2.7466 × 10^1^	9.2824 × 10^1^	1.6464 × 10^1^	1.7244 × 10^3^	5.9704 × 10^3^	4.3904 × 10^1^	4.4268 × 10^3^	2.2560 × 10^3^	7.3596 × 10^1^	4.9290 × 10^1^	4.8649 × 10^2^	7.4718 × 10^0^
F5	mean	6.6823 × 10^2^	7.2608 × 10^2^	7.1876 × 10^2^	7.4565 × 10^2^	1.0537 × 10^3^	7.4869 × 10^2^	1.0185 × 10^3^	7.2777 × 10^2^	7.8766 × 10^2^	7.5841 × 10^2^	7.4342 × 10^2^	6.4080 × 10^2^
	std	2.9983 × 10^1^	4.8066 × 10^1^	1.2375 × 10^1^	4.6566 × 10^1^	4.4448 × 10^1^	3.8245 × 10^1^	3.8090 × 10^1^	3.8033 × 10^1^	4.8540 × 10^1^	3.4278 × 10^1^	3.5956 × 10^1^	1.1530 × 10^1^
F6	mean	6.4100 × 10^2^	6.3615 × 10^2^	6.0054 × 10^2^	6.5268 × 10^2^	7.0926 × 10^2^	6.5970 × 10^2^	7.0524 × 10^2^	6.5855 × 10^2^	6.6671 × 10^2^	6.5749 × 10^2^	6.5761 × 10^2^	6.3587 × 10^2^
	std	8.1053 × 10^0^	1.1999 × 10^1^	1.2669 × 10^−1^	1.1372 × 10^1^	9.0599 × 10^0^	8.3707 × 10^0^	1.1216 × 10^1^	6.0173 × 10^0^	1.0812 × 10^1^	7.4344 × 10^0^	9.2870 × 10^0^	3.7184 × 10^0^
F7	mean	8.4572 × 10^2^	9.4577 × 10^2^	9.6538 × 10^2^	1.1275 × 10^3^	2.7866 × 10^3^	1.1651 × 10^3^	2.6477 × 10^3^	1.1853 × 10^3^	1.3761 × 10^3^	1.1734 × 10^3^	1.1047 × 10^3^	9.1610 × 10^2^
	std	2.7599 × 10^1^	8.5186 × 10^1^	1.0709 × 10^1^	8.1321 × 10^1^	1.4542 × 10^2^	7.8294 × 10^1^	1.8454 × 10^2^	7.2807 × 10^1^	1.5558 × 10^2^	9.8289 × 10^1^	1.0274 × 10^2^	2.8965 × 10^1^
F8	mean	9.2037 × 10^2^	1.0062 × 10^3^	1.0198 × 10^3^	9.9951 × 10^2^	1.2775 × 10^3^	9.8063 × 10^2^	1.2681 × 10^3^	9.6371 × 10^2^	1.0062 × 10^3^	9.8111 × 10^2^	9.9628 × 10^2^	9.0442 × 10^2^
	std	2.2809 × 10^1^	6.1351 × 10^1^	1.2538 × 10^1^	2.7507 × 10^1^	3.4889 × 10^1^	3.0043 × 10^1^	3.8253 × 10^1^	4.3419 × 10^1^	4.7866 × 10^1^	3.3167 × 10^1^	3.0428 × 10^1^	6.3807 × 10^0^
F9	mean	4.4760 × 10^3^	5.5061 × 10^3^	1.1026 × 10^3^	4.8459 × 10^3^	2.5527 × 10^4^	5.9736 × 10^3^	2.2294 × 10^4^	4.7678 × 10^3^	7.8899 × 10^3^	5.7999 × 10^3^	5.3273 × 10^3^	3.1510 × 10^3^
	std	1.1760 × 10^3^	2.0868 × 10^3^	7.0045 × 10^1^	9.2771 × 10^2^	3.1517 × 10^3^	1.3419 × 10^3^	4.0851 × 10^3^	8.0420 × 10^2^	2.0837 × 10^3^	4.6560 × 10^2^	1.0388 × 10^3^	3.0085 × 10^2^
F10	mean	4.7975 × 10^3^	5.3850 × 10^3^	8.3255 × 10^3^	5.6208 × 10^3^	9.4978 × 10^3^	6.2471 × 10^3^	9.1113 × 10^3^	5.0180 × 10^3^	5.0336 × 10^3^	5.1503 × 10^3^	5.8335 × 10^3^	4.3607 × 10^3^
	std	7.3037 × 10^2^	7.6270 × 10^2^	3.7251 × 10^2^	3.9800 × 10^2^	3.7859 × 10^2^	9.1376 × 10^2^	3.4878 × 10^2^	8.6048 × 10^2^	4.7119 × 10^2^	5.7052 × 10^2^	6.3513 × 10^2^	3.3057 × 10^2^
F11	mean	1.2146 × 10^3^	1.5807 × 10^3^	1.3076 × 10^3^	1.6051 × 10^3^	1.8695 × 10^4^	1.6141 × 10^3^	1.8857 × 10^4^	1.3375 × 10^3^	1.2660 × 10^3^	1.4021 × 10^3^	2.0465 × 10^3^	1.2236 × 10^3^
	std	2.9453 × 10^1^	3.0856 × 10^2^	2.3674 × 10^1^	2.1265 × 10^2^	5.4881 × 10^3^	1.9013 × 10^2^	5.6987 × 10^3^	2.5045 × 10^2^	4.3000 × 10^1^	4.2434 × 10^1^	6.9747 × 10^2^	2.4300 × 10^1^
F12	mean	1.0111 × 10^6^	2.5295 × 10^7^	1.0060 × 10^7^	2.8625 × 10^8^	1.3365 × 10^10^	1.2902 × 10^8^	1.1257 × 10^10^	4.3250 × 10^8^	1.3944 × 10^6^	7.6292 × 10^7^	1.4023 × 10^8^	4.3800 × 10^6^
	std	4.5521 × 10^5^	3.5154 × 10^7^	3.5443 × 10^6^	3.6221 × 10^8^	3.3381 × 10^9^	1.2476 × 10^8^	2.8103 × 10^9^	1.0600 × 10^9^	1.2581 × 10^6^	5.5136 × 10^7^	1.3252 × 10^8^	1.6557 × 10^6^
F13	mean	1.8993 × 10^4^	1.6274 × 10^6^	1.4709 × 10^4^	2.9759 × 10^4^	9.7732 × 10^9^	4.6369 × 10^6^	7.1522 × 10^9^	5.5056 × 10^6^	3.9751 × 10^4^	1.0077 × 10^6^	2.1756 × 10^7^	4.1934 × 10^4^
	std	2.0013 × 10^4^	1.9652 × 10^6^	9.3708 × 10^3^	2.2157 × 10^4^	4.2451 × 10^9^	1.3241 × 10^7^	2.4771 × 10^9^	2.1391 × 10^7^	2.3361 × 10^4^	1.7137 × 10^6^	4.9355 × 10^7^	1.2269 × 10^4^
F14	mean	2.8124 × 10^4^	1.3929 × 10^5^	8.1612 × 10^4^	1.5347 × 10^3^	7.3854 × 10^6^	4.3445 × 10^5^	4.6119 × 10^6^	2.4762 × 10^3^	3.2515 × 10^4^	1.0523 × 10^5^	3.1896 × 10^5^	9.2681 × 10^3^
	std	3.4144 × 10^4^	1.6541 × 10^5^	3.6973 × 10^4^	3.4674 × 10^1^	4.8434 × 10^6^	4.1255 × 10^5^	3.2202 × 10^6^	1.3168 × 10^3^	2.9287 × 10^4^	6.9809 × 10^4^	3.7534 × 10^5^	3.1857 × 10^3^
F15	mean	7.8991 × 10^3^	8.5189 × 10^4^	3.7989 × 10^3^	3.0194 × 10^3^	1.7927 × 10^9^	1.5656 × 10^5^	9.1793 × 10^8^	3.0543 × 10^4^	1.4028 × 10^4^	4.7496 × 10^4^	5.4897 × 10^5^	1.1367 × 10^4^
	std	7.2737 × 10^3^	9.4273 × 10^4^	2.9766 × 10^3^	1.0039 × 10^3^	1.1773 × 10^9^	2.4076 × 10^5^	5.6871 × 10^8^	3.7296 × 10^4^	1.3247 × 10^4^	3.9716 × 10^4^	1.4112 × 10^6^	3.1207 × 10^3^
F16	mean	2.7016 × 10^3^	3.1543 × 10^3^	3.2198 × 10^3^	2.5685 × 10^3^	5.5876 × 10^3^	3.6855 × 10^3^	5.2419 × 10^3^	2.9788 × 10^3^	3.1881 × 10^3^	2.9683 × 10^3^	3.2435 × 10^3^	2.5947 × 10^3^
	std	2.5256 × 10^2^	4.4247 × 10^2^	1.7290 × 10^2^	2.9293 × 10^2^	6.0550 × 10^2^	4.7665 × 10^2^	5.2355 × 10^2^	4.4153 × 10^2^	3.8850 × 10^2^	3.6159 × 10^2^	3.3138 × 10^2^	1.2817 × 10^2^
F17	mean	2.2794 × 10^3^	2.5730 × 10^3^	2.3092 × 10^3^	1.9461 × 10^3^	4.1238 × 10^3^	2.4383 × 10^3^	3.6327 × 10^3^	2.2426 × 10^3^	2.6080 × 10^3^	2.3038 × 10^3^	2.3449 × 10^3^	1.9846 × 10^3^
	std	2.7717 × 10^2^	2.5420 × 10^2^	1.2729 × 10^2^	1.0835 × 10^2^	8.1611 × 10^2^	2.2300 × 10^2^	3.5718 × 10^2^	1.7419 × 10^2^	2.4774 × 10^2^	2.3595 × 10^2^	1.9392 × 10^2^	8.0074 × 10^1^
F18	mean	4.4829 × 10^5^	1.1853 × 10^6^	3.6019 × 10^6^	4.7945 × 10^3^	6.5949 × 10^7^	2.2040 × 10^6^	4.3159 × 10^7^	1.6409 × 10^5^	2.7332 × 10^5^	1.5461 × 10^6^	1.3844 × 10^6^	1.5971 × 10^5^
	std	4.0547 × 10^5^	1.2893 × 10^6^	1.7371 × 10^6^	5.1017 × 10^3^	3.7026 × 10^7^	1.6357 × 10^6^	3.4046 × 10^7^	4.8933 × 10^5^	2.0796 × 10^5^	1.4657 × 10^6^	1.5346 × 10^6^	4.9887 × 10^4^
F19	mean	8.5089 × 10^3^	4.9179 × 10^5^	7.7215 × 10^3^	2.4706 × 10^3^	1.8294 × 10^9^	3.8168 × 10^6^	1.1551 × 10^9^	2.9972 × 10^5^	1.2667 × 10^4^	6.8433 × 10^5^	1.4214 × 10^6^	1.0679 × 10^5^
	std	7.0987 × 10^3^	9.8238 × 10^5^	6.0219 × 10^3^	1.0744 × 10^3^	7.6056 × 10^8^	2.5128 × 10^6^	4.4618 × 10^8^	6.6506 × 10^5^	1.2903 × 10^4^	9.0218 × 10^5^	2.4026 × 10^6^	7.3105 × 10^4^
F20	mean	2.5483 × 10^3^	2.7128 × 10^3^	2.6962 × 10^3^	2.3145 × 10^3^	3.2669 × 10^3^	2.6522 × 10^3^	3.1480 × 10^3^	2.5114 × 10^3^	2.7583 × 10^3^	2.6160 × 10^3^	2.6266 × 10^3^	2.3468 × 10^3^
	std	1.7608 × 10^2^	2.1449 × 10^2^	9.7777 × 10^1^	7.1269 × 10^1^	1.4208 × 10^2^	1.6342 × 10^2^	1.3700 × 10^2^	1.8423 × 10^2^	2.1798 × 10^2^	1.8945 × 10^2^	1.7636 × 10^2^	3.6869 × 10^1^
F21	mean	2.4721 × 10^3^	2.5349 × 10^3^	2.5125 × 10^3^	2.4735 × 10^3^	2.7991 × 10^3^	2.5409 × 10^3^	2.7590 × 10^3^	2.5150 × 10^3^	2.5705 × 10^3^	2.5222 × 10^3^	2.5184 × 10^3^	2.4203 × 10^3^
	std	3.1714 × 10^1^	3.7455 × 10^1^	1.1836 × 10^1^	6.5982 × 10^1^	4.1707 × 10^1^	4.2051 × 10^1^	4.1855 × 10^1^	5.5133 × 10^1^	4.1515 × 10^1^	3.9185 × 10^1^	4.9359 × 10^1^	1.1352 × 10^1^
F22	mean	4.3993 × 10^3^	5.4299 × 10^3^	4.5365 × 10^3^	4.8925 × 10^3^	1.0689 × 10^4^	3.7756 × 10^3^	9.9037 × 10^3^	5.7577 × 10^3^	5.8230 × 10^3^	4.5446 × 10^3^	3.8712 × 10^3^	2.3124 × 10^3^
	std	2.1899 × 10^3^	2.0346 × 10^3^	2.4460 × 10^3^	8.0687 × 10^2^	5.7949 × 10^2^	2.1091 × 10^3^	1.2695 × 10^3^	2.0636 × 10^3^	2.0755 × 10^3^	2.5788 × 10^3^	1.5267 × 10^3^	1.3613
F23	mean	3.1413 × 10^3^	2.9146 × 10^3^	2.8537 × 10^3^	2.9144 × 10^3^	3.5201 × 10^3^	2.9968 × 10^3^	3.3742 × 10^3^	3.0377 × 10^3^	3.0621 × 10^3^	2.9631 × 10^3^	2.9316 × 10^3^	2.7885 × 10^3^
	std	1.3554 × 10^2^	6.4965 × 10^1^	1.3865 × 10^1^	4.7353 × 10^1^	1.0623 × 10^2^	9.2096 × 10^1^	6.5209 × 10^1^	1.1706 × 10^2^	1.2011 × 10^2^	6.8163 × 10^1^	6.3598 × 10^1^	1.3490 × 10^1^
F24	mean	3.1804 × 10^3^	3.0670 × 10^3^	3.0217 × 10^3^	3.0915 × 10^3^	3.7703 × 10^3^	3.1479 × 10^3^	3.5671 × 10^3^	3.1898 × 10^3^	3.2614 × 10^3^	3.1565 × 10^3^	3.0356 × 10^3^	2.9463 × 10^3^
	std	6.9984 × 10^1^	7.0714 × 10^1^	1.3135 × 10^1^	7.0599 × 10^1^	1.7933 × 10^2^	7.9792 × 10^1^	1.0491 × 10^2^	1.0203 × 10^2^	9.2964 × 10^1^	6.6042 × 10^1^	5.2799 × 10^1^	1.8954 × 10^1^
F25	mean	2.8868 × 10^3^	2.9437 × 10^3^	2.8892 × 10^3^	3.5497 × 10^3^	1.0953 × 10^4^	2.9960 × 10^3^	9.9811 × 10^3^	3.0164 × 10^3^	2.9273 × 10^3^	3.0173 × 10^3^	3.0673 × 10^3^	2.8980 × 10^3^
	std	1.6211 × 10^1^	3.7801 × 10^1^	9.0494 × 10^−1^	2.8154 × 10^2^	1.9415 × 10^3^	3.2839 × 10^1^	1.5360 × 10^3^	2.6358 × 10^2^	2.4654 × 10^1^	5.0787 × 10^1^	7.2889 × 10^1^	7.4671
F26	mean	6.0316 × 10^3^	6.2387 × 10^3^	5.7669 × 10^3^	7.0490 × 10^3^	1.2366 × 10^4^	6.7362 × 10^3^	1.1209 × 10^4^	7.3263 × 10^3^	7.1320 × 10^3^	6.2685 × 10^3^	6.1536 × 10^3^	2.8834 × 10^3^
	std	1.8462 × 10^3^	1.0046 × 10^3^	1.1892 × 10^2^	1.2078 × 10^3^	9.3537 × 10^2^	1.4815 × 10^3^	9.8286 × 10^2^	1.3198 × 10^3^	7.7884 × 10^2^	1.6524 × 10^3^	1.1082 × 10^3^	6.1073 × 10^1^
F27	mean	3.3207 × 10^3^	3.2832 × 10^3^	3.2265 × 10^3^	3.3037 × 10^3^	4.2525 × 10^3^	3.3950 × 10^3^	3.9930 × 10^3^	3.3906 × 10^3^	3.4781 × 10^3^	3.3330 × 10^3^	3.3631 × 10^3^	3.2553 × 10^3^
	std	1.8663 × 10^2^	4.2780 × 10^1^	5.2334 × 10^0^	8.2012 × 10^1^	3.1731 × 10^2^	8.0192 × 10^1^	1.6880 × 10^2^	1.1327 × 10^2^	1.5062 × 10^2^	7.9206 × 10^1^	7.4251 × 10^1^	1.0003 × 10^1^
F28	mean	3.2273 × 10^3^	3.4576 × 10^3^	3.2813 × 10^3^	4.5518 × 10^3^	9.2000 × 10^3^	3.3852 × 10^3^	8.5148 × 10^3^	3.5787 × 10^3^	3.2535 × 10^3^	3.3838 × 10^3^	3.4963 × 10^3^	3.2263 × 10^3^
	std	2.5873 × 10^1^	4.1676 × 10^2^	1.7738 × 10^1^	5.4461 × 10^2^	1.1052 × 10^3^	5.8650 × 10^1^	7.6068 × 10^2^	7.5970 × 10^2^	2.1426 × 10^1^	5.8545 × 10^1^	9.7327 × 10^1^	9.1916
F29	mean	4.1318 × 10^3^	4.1407 × 10^3^	4.2951 × 10^3^	4.0358 × 10^3^	7.4570 × 10^3^	4.8503 × 10^3^	6.5519 × 10^3^	4.4184 × 10^3^	4.3611 × 10^3^	4.2380 × 10^3^	4.6267 × 10^3^	4.0188 × 10^3^
	std	2.3381 × 10^2^	3.0088 × 10^2^	1.6788 × 10^2^	2.1658 × 10^2^	1.5567 × 10^3^	4.4456 × 10^2^	6.6381 × 10^2^	3.4373 × 10^2^	2.8790 × 10^2^	3.0932 × 10^2^	3.7478 × 10^2^	9.3907 × 10^1^
F30	mean	4.6429 × 10^4^	1.7286 × 10^6^	1.7652 × 10^5^	9.8724 × 10^4^	1.1312 × 10^9^	2.8170 × 10^7^	8.8037 × 10^8^	1.9250 × 10^6^	1.1450 × 10^5^	6.7548 × 10^6^	1.2843 × 10^7^	1.5369 × 10^6^
	std	2.9880 × 10^4^	2.6549 × 10^6^	1.1343 × 10^5^	1.5524 × 10^5^	6.3220 × 10^8^	4.0626 × 10^7^	4.9386 × 10^8^	3.1153 × 10^6^	8.5736 × 10^4^	5.7149 × 10^6^	1.1160 × 10^7^	5.4004 × 10^5^

**Table 3:** Results of various algorithms tested on the CEC 2017 benchmark (dim = 50).

ID	Metric	PSO	DBO	DMO	IAO	MGO	PO	PLO	BKA	CPSOGSA	HPHHO	SCSO	MISCSO
F1	mean	8.9897 × 10^6^	3.8830 × 10^8^	6.8241 × 10^8^	7.2852 × 10^10^	2.0034 × 10^11^	3.0362 × 10^9^	1.8587 × 10^11^	1.3892 × 10^10^	8.0228 × 10^7^	1.2151 × 10^10^	1.6462 × 10^10^	1.7401 × 10^7^
	std	3.0397 × 10^6^	2.0759 × 10^8^	1.6897 × 10^8^	1.1916 × 10^10^	1.7560 × 10^10^	1.6281 × 10^9^	1.7736 × 10^10^	1.3333 × 10^10^	3.1379 × 10^8^	2.9029 × 10^9^	4.5194 × 10^9^	3.1662 × 10^6^
F2	mean	1.7366 × 10^23^	4.9106 × 10^59^	2.5265 × 10^65^	2.5978 × 10^67^	4.5250 × 10^85^	6.3464 × 10^55^	1.6571 × 10^81^	1.1868 × 10^70^	7.5540 × 10^57^	1.0000 × 10^20^	1.7256 × 10^61^	1.5470 × 10^35^
	std	6.0879 × 10^23^	1.7983 × 10^60^	7.1325 × 10^65^	1.4213 × 10^68^	2.0008 × 10^86^	3.0826 × 10^56^	4.7047 × 10^81^	6.5005 × 10^70^	4.0307 × 10^58^	0.0000 × 10^0^	9.4203 × 10^61^	2.0672 × 10^35^
F3	mean	7.6456 × 10^4^	2.1162 × 10^5^	3.0074 × 10^5^	1.0315 × 10^5^	4.4547 × 10^5^	1.3993 × 10^5^	3.9858 × 10^5^	6.1453 × 10^4^	2.3049 × 10^5^	7.8358 × 10^4^	1.0695 × 10^5^	5.0798 × 10^4^
	std	2.2408 × 10^4^	4.5391 × 10^4^	2.8965 × 10^4^	1.8764 × 10^4^	1.1217 × 10^5^	1.9049 × 10^4^	5.1826 × 10^4^	3.8998 × 10^4^	5.8799 × 10^4^	1.0846 × 10^4^	1.8706 × 10^4^	4.2001 × 10^3^
F4	mean	5.3716 × 10^2^	8.0239 × 10^2^	8.7736 × 10^2^	1.8250 × 10^4^	7.0239 × 10^4^	1.0449 × 10^3^	5.5375 × 10^4^	3.1949 × 10^3^	6.4201 × 10^2^	1.9547 × 10^3^	2.4143 × 10^3^	5.6831 × 10^2^
	std	5.0765 × 10^1^	1.2802 × 10^2^	5.5883 × 10^1^	5.6579 × 10^3^	1.2618 × 10^4^	2.1039 × 10^2^	1.2311 × 10^4^	5.6780 × 10^3^	5.7264 × 10^1^	6.7531 × 10^2^	1.1714 × 10^3^	3.3318 × 10^1^
F5	mean	7.6352 × 10^2^	9.4542 × 10^2^	9.7494 × 10^2^	9.9920 × 10^2^	1.5085 × 10^3^	9.1577 × 10^2^	1.4719 × 10^3^	8.7416 × 10^2^	9.7727 × 10^2^	9.3596 × 10^2^	9.1821 × 10^2^	7.6681 × 10^2^
	std	3.2423 × 10^1^	8.4692 × 10^1^	1.7387 × 10^1^	3.6338 × 10^1^	6.1268 × 10^1^	4.3380 × 10^1^	5.6923 × 10^1^	6.6440 × 10^1^	7.5410 × 10^1^	3.3968 × 10^1^	3.8691 × 10^1^	1.2256 × 10^1^
F6	mean	6.5033 × 10^2^	6.5599 × 10^2^	6.1348 × 10^2^	6.6854 × 10^2^	7.2929 × 10^2^	6.7630 × 10^2^	7.2743 × 10^2^	6.6713 × 10^2^	6.7526 × 10^2^	6.7319 × 10^2^	6.7171 × 10^2^	6.5399 × 10^2^
	std	5.6599 × 10^0^	1.1148 × 10^1^	1.7602 × 10^0^	8.5585 × 10^0^	1.0039 × 10^1^	6.9614 × 10^0^	7.6965 × 10^0^	6.3200 × 10^0^	9.1079 × 10^0^	6.6246 × 10^0^	6.7441 × 10^0^	3.2413 × 10^0^
F7	mean	1.0948 × 10^3^	1.2719 × 10^3^	1.2956 × 10^3^	1.6689 × 10^3^	5.0273 × 10^3^	1.6449 × 10^3^	5.0203 × 10^3^	1.6836 × 10^3^	2.3661 × 10^3^	1.6965 × 10^3^	1.6117 × 10^3^	1.2508 × 10^3^
	std	8.8706 × 10^1^	1.7884 × 10^2^	2.8861 × 10^1^	1.1367 × 10^2^	3.0848 × 10^2^	1.0492 × 10^2^	3.9740 × 10^2^	8.4073 × 10^1^	2.7540 × 10^2^	1.2634 × 10^2^	1.2786 × 10^2^	5.6057 × 10^1^
F8	mean	1.0851 × 10^3^	1.2816 × 10^3^	1.2715 × 10^3^	1.3117 × 10^3^	1.8246 × 10^3^	1.2461 × 10^3^	1.7768 × 10^3^	1.2152 × 10^3^	1.2208 × 10^3^	1.2429 × 10^3^	1.2261 × 10^3^	1.0789 × 10^3^
	std	3.3980 × 10^1^	8.4717 × 10^1^	1.8896 × 10^1^	3.9585 × 10^1^	8.4306 × 10^1^	4.0751 × 10^1^	5.2736 × 10^1^	8.0004 × 10^1^	6.1311 × 10^1^	3.4434 × 10^1^	4.4748 × 10^1^	1.9967 × 10^1^
F9	mean	2.2091 × 10^4^	1.6831 × 10^4^	6.8065 × 10^3^	2.0580 × 10^4^	7.5428 × 10^4^	2.2588 × 10^4^	6.8868 × 10^4^	1.5273 × 10^4^	1.9327 × 10^4^	1.9931 × 10^4^	1.9139 × 10^4^	1.1559 × 10^4^
	std	4.4515 × 10^3^	6.8780 × 10^3^	1.4345 × 10^3^	2.6027 × 10^3^	8.0130 × 10^3^	2.6779 × 10^3^	8.1827 × 10^3^	3.7172 × 10^3^	3.9444 × 10^3^	3.0756 × 10^3^	2.9953 × 10^3^	1.4641 × 10^3^
F10	mean	7.5691 × 10^3^	9.4782 × 10^3^	1.4822 × 10^4^	1.0629 × 10^4^	1.6027 × 10^4^	1.1254 × 10^4^	1.5770 × 10^4^	9.3151 × 10^3^	8.2329 × 10^3^	9.5573 × 10^3^	9.7088 × 10^3^	7.3454 × 10^3^
	std	1.0257 × 10^3^	1.6171 × 10^3^	3.8813 × 10^2^	6.7649 × 10^2^	7.1636 × 10^2^	1.1608 × 10^3^	4.8646 × 10^2^	2.0822 × 10^3^	7.2495 × 10^2^	1.0361 × 10^3^	8.8439 × 10^2^	5.1975 × 10^2^
F11	mean	1.3084 × 10^3^	2.8057 × 10^3^	3.4290 × 10^3^	8.4334 × 10^3^	5.0425 × 10^4^	3.4656 × 10^3^	5.0020 × 10^4^	2.1447 × 10^3^	1.6932 × 10^3^	2.0302 × 10^3^	5.3820 × 10^3^	1.4838 × 10^3^
	std	4.4726 × 10^1^	3.1472 × 10^3^	6.6895 × 10^2^	2.5305 × 10^3^	9.6248 × 10^3^	8.4350 × 10^2^	8.9463 × 10^3^	8.1748 × 10^2^	2.1881 × 10^2^	2.1977 × 10^2^	2.2845 × 10^3^	4.1901 × 10^1^
F12	mean	1.2636 × 10^7^	4.4924 × 10^8^	4.0077 × 10^8^	1.9878 × 10^10^	8.3546 × 10^10^	6.4397 × 10^8^	7.2780 × 10^10^	5.2672 × 10^9^	2.8980 × 10^7^	7.4617 × 10^8^	2.3667 × 10^9^	4.3955 × 10^7^
	std	6.8351 × 10^6^	4.3211 × 10^8^	9.2467 × 10^7^	8.4191 × 10^9^	1.8339 × 10^10^	3.2408 × 10^8^	1.2532 × 10^10^	1.1417 × 10^10^	1.6236 × 10^7^	5.5954 × 10^8^	1.7902 × 10^9^	1.5910 × 10^7^
F13	mean	3.1687 × 10^4^	1.3667 × 10^7^	6.6872 × 10^3^	4.7262 × 10^8^	4.1161 × 10^10^	6.6942 × 10^7^	3.5808 × 10^10^	1.4816 × 10^9^	6.6906 × 10^4^	2.3602 × 10^7^	1.7187 × 10^8^	1.3468 × 10^5^
	std	1.6727 × 10^4^	1.6969 × 10^7^	4.1016 × 10^3^	6.3704 × 10^8^	1.2017 × 10^10^	9.7544 × 10^7^	9.5093 × 10^9^	3.9241 × 10^9^	4.7047 × 10^4^	2.7145 × 10^7^	2.3588 × 10^8^	2.6952 × 10^4^
F14	mean	1.3470 × 10^5^	2.1477 × 10^6^	1.5048 × 10^6^	2.0190 × 10^3^	7.4678 × 10^7^	3.0796 × 10^6^	4.4965 × 10^7^	1.0076 × 10^5^	2.3467 × 10^5^	1.1184 × 10^6^	1.3126 × 10^6^	1.8089 × 10^5^
	std	9.3046 × 10^4^	2.1338 × 10^6^	5.8449 × 10^5^	4.5436 × 10^2^	6.0237 × 10^7^	1.6255 × 10^6^	2.8857 × 10^7^	1.7383 × 10^5^	1.6995 × 10^5^	7.7343 × 10^5^	1.6067 × 10^6^	6.1229 × 10^4^
F15	mean	9.4583 × 10^3^	4.1022 × 10^7^	1.0580 × 10^4^	2.4265 × 10^4^	1.4440 × 10^10^	3.7018 × 10^6^	1.0244 × 10^10^	1.3835 × 10^8^	3.3724 × 10^4^	2.2098 × 10^6^	3.5576 × 10^7^	2.5660 × 10^4^
	std	7.4309 × 10^3^	1.6150 × 10^8^	4.1280 × 10^3^	1.4673 × 10^4^	5.0160 × 10^9^	5.7302 × 10^6^	3.2543 × 10^9^	3.5153 × 10^8^	1.6147 × 10^4^	3.2240 × 10^6^	1.1387 × 10^8^	4.2022 × 10^3^
F16	mean	3.3922 × 10^3^	4.5915 × 10^3^	5.0723 × 10^3^	4.1689 × 10^3^	9.4577 × 10^3^	4.7916 × 10^3^	8.6668 × 10^3^	4.1650 × 10^3^	4.0791 × 10^3^	4.2674 × 10^3^	4.4603 × 10^3^	3.4372 × 10^3^
	std	4.9670 × 10^2^	5.9073 × 10^2^	2.3257 × 10^2^	4.4128 × 10^2^	1.0670 × 10^3^	7.3801 × 10^2^	8.1791 × 10^2^	9.4155 × 10^2^	5.4438 × 10^2^	5.6211 × 10^2^	5.2932 × 10^2^	2.3198 × 10^2^
F17	mean	3.1624 × 10^3^	4.0404 × 10^3^	3.9932 × 10^3^	3.1527 × 10^3^	6.2401 × 10^4^	3.9160 × 10^3^	3.4947 × 10^4^	3.4769 × 10^3^	3.8316 × 10^3^	3.5956 × 10^3^	3.7673 × 10^3^	3.0168 × 10^3^
	std	3.5573 × 10^2^	4.9686 × 10^2^	1.5217 × 10^2^	2.3741 × 10^2^	6.6416 × 10^4^	3.9606 × 10^2^	4.0201 × 10^4^	4.6386 × 10^2^	4.9900 × 10^2^	3.3314 × 10^2^	4.1176 × 10^2^	1.5959 × 10^2^
F18	mean	1.4819 × 10^6^	6.1166 × 10^6^	1.6441 × 10^7^	8.8023 × 10^4^	2.5618 × 10^8^	1.2051 × 10^7^	1.7325 × 10^8^	3.1260 × 10^6^	1.2420 × 10^6^	5.8020 × 10^6^	1.1772 × 10^7^	8.2573 × 10^5^
	std	1.0028 × 10^6^	6.3179 × 10^6^	6.0826 × 10^6^	3.8600 × 10^4^	1.3547 × 10^8^	1.0071 × 10^7^	6.4040 × 10^7^	9.7107 × 10^6^	1.1660 × 10^6^	4.8214 × 10^6^	1.5287 × 10^7^	2.4819 × 10^5^
F19	mean	1.5635 × 10^4^	4.4341 × 10^6^	1.6262 × 10^4^	3.3861 × 10^5^	5.5993 × 10^9^	7.8509 × 10^6^	4.2829 × 10^9^	5.0375 × 10^7^	2.6380 × 10^4^	7.0202 × 10^5^	8.4308 × 10^6^	3.5103 × 10^5^
	std	1.0625 × 10^4^	6.9827 × 10^6^	6.8507 × 10^3^	5.0725 × 10^5^	1.5878 × 10^9^	6.7613 × 10^6^	1.2356 × 10^9^	1.5638 × 10^8^	1.6900 × 10^4^	7.7571 × 10^5^	1.1979 × 10^7^	2.1807 × 10^5^
F20	mean	3.1615 × 10^3^	3.5236 × 10^3^	4.0469 × 10^3^	2.8847 × 10^3^	4.7552 × 10^3^	3.5465 × 10^3^	4.7079 × 10^3^	3.2162 × 10^3^	3.4197 × 10^3^	3.1248 × 10^3^	3.3806 × 10^3^	2.9304 × 10^3^
	std	3.4935 × 10^2^	3.6323 × 10^2^	2.1201 × 10^2^	1.5020 × 10^2^	2.7404 × 10^2^	3.2637 × 10^2^	2.8110 × 10^2^	2.5417 × 10^2^	3.1547 × 10^2^	2.8352 × 10^2^	2.6095 × 10^2^	1.0680 × 10^2^
F21	mean	2.6717 × 10^3^	2.7804 × 10^3^	2.7634 × 10^3^	2.8274 × 10^3^	3.3412 × 10^3^	2.7984 × 10^3^	3.2658 × 10^3^	2.7986 × 10^3^	2.8834 × 10^3^	2.8095 × 10^3^	2.7442 × 10^3^	2.5964 × 10^3^
	std	5.9562 × 10^1^	7.0692 × 10^1^	1.8472 × 10^1^	4.9625 × 10^1^	6.4493 × 10^1^	7.3872 × 10^1^	6.7888 × 10^1^	8.3227 × 10^1^	9.5616 × 10^1^	6.9845 × 10^1^	6.6961 × 10^1^	2.1165 × 10^1^
F22	mean	9.2216 × 10^3^	1.1527 × 10^4^	1.6071 × 10^4^	1.2242 × 10^4^	1.7902 × 10^4^	1.2663 × 10^4^	1.7281 × 10^4^	9.9427 × 10^3^	1.0028 × 10^4^	1.2031 × 10^4^	1.1786 × 10^4^	8.4733 × 10^3^
	std	9.5078 × 10^2^	1.3529 × 10^3^	1.0737 × 10^3^	1.2702 × 10^3^	4.7019 × 10^2^	1.2700 × 10^3^	5.6768 × 10^2^	8.9377 × 10^2^	9.3330 × 10^2^	9.1668 × 10^2^	1.1439 × 10^3^	2.1008 × 10^3^
F23	mean	3.5916 × 10^3^	3.3896 × 10^3^	3.1828 × 10^3^	3.4926 × 10^3^	4.4736 × 10^3^	3.5000 × 10^3^	4.2168 × 10^3^	3.7011 × 10^3^	3.5119 × 10^3^	3.4746 × 10^3^	3.2987 × 10^3^	3.1267 × 10^3^
	std	2.2505 × 10^2^	1.1927 × 10^2^	1.7076 × 10^1^	1.3328 × 10^2^	2.4277 × 10^2^	1.2086 × 10^2^	1.4020 × 10^2^	1.8246 × 10^2^	1.1937 × 10^2^	1.5604 × 10^2^	8.8678 × 10^1^	2.1117 × 10^1^
F24	mean	3.5469 × 10^3^	3.4724 × 10^3^	3.3312 × 10^3^	3.5904 × 10^3^	4.9357 × 10^3^	3.6792 × 10^3^	4.5729 × 10^3^	3.7385 × 10^3^	3.9314 × 10^3^	3.6763 × 10^3^	3.4205 × 10^3^	3.2304 × 10^3^
	std	1.2528 × 10^2^	1.0877 × 10^2^	1.2478 × 10^1^	9.1181 × 10^1^	2.4920 × 10^2^	1.4087 × 10^2^	1.5763 × 10^2^	1.2822 × 10^2^	1.6048 × 10^2^	1.3779 × 10^2^	9.0690 × 10^1^	2.7000 × 10^1^
F25	mean	2.9835 × 10^3^	3.2277 × 10^3^	3.3276 × 10^3^	1.0425 × 10^4^	3.9095 × 10^4^	3.5450 × 10^3^	3.5369 × 10^4^	4.5997 × 10^3^	3.1239 × 10^3^	3.9349 × 10^3^	4.3439 × 10^3^	3.1040 × 10^3^
	std	4.3010 × 10^1^	8.7647 × 10^1^	6.4777 × 10^1^	1.3513 × 10^3^	5.6212 × 10^3^	1.7605 × 10^2^	5.3989 × 10^3^	2.4586 × 10^3^	3.2608 × 10^1^	3.0082 × 10^2^	5.1200 × 10^2^	2.1872 × 10^1^
F26	mean	8.4219 × 10^3^	1.0059 × 10^4^	8.3198 × 10^3^	1.3926 × 10^4^	2.3770 × 10^4^	9.5870 × 10^3^	2.0979 × 10^4^	1.1074 × 10^4^	1.1839 × 10^4^	1.1393 × 10^4^	1.0844 × 10^4^	3.7741 × 10^3^
	std	3.4611 × 10^3^	1.0294 × 10^3^	2.0570 × 10^2^	1.0432 × 10^3^	2.2506 × 10^3^	2.5757 × 10^3^	1.8586 × 10^3^	2.2617 × 10^3^	1.3737 × 10^3^	1.3204 × 10^3^	2.2053 × 10^3^	3.7862 × 10^2^
F27	mean	3.8664 × 10^3^	3.7775 × 10^3^	3.5385 × 10^3^	3.8716 × 10^3^	6.6507 × 10^3^	4.0439 × 10^3^	5.9407 × 10^3^	4.1983 × 10^3^	4.6542 × 10^3^	3.9183 × 10^3^	4.1476 × 10^3^	3.6785 × 10^3^
	std	6.8506 × 10^2^	1.9470 × 10^2^	3.7385 × 10^1^	1.1793 × 10^2^	4.7349 × 10^2^	3.0257 × 10^2^	3.5751 × 10^2^	3.9154 × 10^2^	3.2811 × 10^2^	2.4541 × 10^2^	2.2826 × 10^2^	5.6679 × 10^1^
F28	mean	3.2911 × 10^3^	5.3736 × 10^3^	3.7746 × 10^3^	8.5576 × 10^3^	1.8003 × 10^4^	4.1462 × 10^3^	1.6527 × 10^4^	4.6413 × 10^3^	3.4260 × 10^3^	4.6468 × 10^3^	5.0102 × 10^3^	3.3727 × 10^3^
	std	3.1086 × 10^1^	2.2895 × 10^3^	1.3090 × 10^2^	8.5361 × 10^2^	1.8625 × 10^3^	2.2770 × 10^2^	1.3132 × 10^3^	1.2330 × 10^3^	1.1099 × 10^2^	3.4522 × 10^2^	4.9098 × 10^2^	1.7543 × 10^1^
F29	mean	5.1661 × 10^3^	5.7056 × 10^3^	5.6765 × 10^3^	6.5253 × 10^3^	7.6935 × 10^4^	7.2752 × 10^3^	4.5461 × 10^4^	6.1550 × 10^3^	6.3027 × 10^3^	6.4383 × 10^3^	6.4535 × 10^3^	5.3625 × 10^3^
	std	3.5375 × 10^2^	8.6028 × 10^2^	2.6418 × 10^2^	1.0053 × 10^3^	6.7638 × 10^4^	9.4449 × 10^2^	2.5702 × 10^4^	1.2094 × 10^3^	5.5447 × 10^2^	7.1051 × 10^2^	8.0067 × 10^2^	2.1798 × 10^2^
F30	mean	4.5567 × 10^6^	2.9449 × 10^7^	1.6845 × 10^7^	6.0409 × 10^7^	7.9246 × 10^9^	2.0830 × 10^8^	6.7389 × 10^9^	1.0056 × 10^8^	4.1553 × 10^7^	1.4806 × 10^8^	1.6542 × 10^8^	6.7228 × 10^7^
	std	1.5213 × 10^6^	2.3182 × 10^7^	7.0009 × 10^6^	3.1111 × 10^7^	2.9827 × 10^9^	8.3280 × 10^7^	2.9003 × 10^9^	3.0905 × 10^8^	1.6847 × 10^7^	5.7281 × 10^7^	1.2992 × 10^8^	1.0398 × 10^7^

**Table 4:** Results of various algorithms tested on the CEC 2017 benchmark (dim = 100).

ID	Metric	PSO	DBO	DMO	IAO	MGO	PO	PLO	BKA	CPSOGSA	HPHHO	SCSO	MISCSO
F1	mean	1.3417 × 10^8^	7.5275 × 10^10^	5.3144 × 10^10^	2.1959 × 10^11^	5.2807 × 10^11^	2.4302 × 10^10^	5.1969 × 10^11^	9.9858 × 10^10^	2.6018 × 10^9^	6.8836 × 10^10^	7.1122 × 10^10^	5.9830 × 10^8^
	std	3.0715 × 10^7^	7.1062 × 10^10^	4.5607 × 10^9^	1.9282 × 10^10^	3.1059 × 10^10^	5.1713 × 10^9^	3.9216 × 10^10^	3.7310 × 10^10^	1.4501 × 10^9^	1.3717 × 10^10^	1.5200 × 10^10^	8.6889 × 10^7^
F2	mean	3.4896 × 10^70^	7.6065 × 10^149^	1.0263 × 10^154^	4.6125 × 10^153^	1.5353 × 10^184^	2.0033 × 10^147^	7.7085 × 10^180^	8.5159 × 10^159^	1.0393 × 10^144^	1.0000 × 10^20^	3.1511 × 10^147^	3.9429 × 10^106^
	std	1.8584 × 10^71^	4.1657 × 10^150^	6.5535 × 10^4^	6.5535 × 10^4^	6.5535 × 10^4^	1.0931 × 10^148^	6.5535 × 10^4^	6.5535 × 10^4^	4.1438 × 10^144^	0.0000 × 10^0^	1.2700 × 10^148^	6.4596 × 10^106^
F3	mean	3.8388 × 10^5^	5.1027 × 10^5^	7.9320 × 10^5^	2.7192 × 10^5^	7.1314 × 10^6^	3.2742 × 10^5^	9.6133 × 10^5^	2.1070 × 10^5^	7.1606 × 10^5^	2.6138 × 10^5^	2.7788 × 10^5^	2.4673 × 10^5^
	std	6.5156 × 10^4^	1.9002 × 10^5^	5.5838 × 10^4^	2.2068 × 10^4^	3.2253 × 10^7^	1.7737 × 10^4^	1.1674 × 10^5^	4.7311 × 10^4^	1.0491 × 10^5^	2.3532 × 10^4^	1.9801 × 10^4^	9.3426 × 10^3^
F4	mean	7.2547 × 10^2^	7.3972 × 10^3^	6.8730 × 10^3^	6.6603 × 10^4^	2.2101 × 10^5^	3.7932 × 10^3^	1.9902 × 10^5^	1.8181 × 10^4^	1.7090 × 10^3^	9.1244 × 10^3^	8.5617 × 10^3^	1.0729 × 10^3^
	std	7.8092 × 10^1^	7.3116 × 10^3^	9.7090 × 10^2^	1.2471 × 10^4^	2.7481 × 10^4^	8.6808 × 10^2^	2.5537 × 10^4^	2.1043 × 10^4^	4.1576 × 10^2^	2.1043 × 10^3^	2.2072 × 10^3^	4.6174 × 10^1^
F5	mean	1.2616 × 10^3^	1.6985 × 10^3^	1.7317 × 10^3^	1.8512 × 10^3^	2.8740 × 10^3^	1.6066 × 10^3^	2.8265 × 10^3^	1.4397 × 10^3^	1.7515 × 10^3^	1.6633 × 10^3^	1.5491 × 10^3^	1.2647 × 10^3^
	std	6.3621 × 10^1^	2.1028 × 10^2^	3.4889 × 10^1^	4.4516 × 10^1^	1.2735 × 10^2^	5.9595 × 10^1^	9.7750 × 10^1^	9.4815 × 10^1^	1.1151 × 10^2^	6.3565 × 10^1^	6.1464 × 10^1^	2.2101 × 10^1^
F6	mean	6.6425 × 10^2^	6.7480 × 10^2^	6.5356 × 10^2^	6.8974 × 10^2^	7.4996 × 10^2^	6.8707 × 10^2^	7.4546 × 10^2^	6.7519 × 10^2^	6.7767 × 10^2^	6.8418 × 10^2^	6.8075 × 10^2^	6.6850 × 10^2^
	std	4.0104 × 10^0^	1.0430 × 10^1^	2.9298 × 10^0^	3.5934 × 10^0^	7.2824 × 10^0^	4.8998 × 10^0^	6.5568 × 10^0^	7.4819 × 10^0^	5.0511 × 10^0^	3.6326 × 10^0^	5.2711 × 10^0^	1.4058 × 10^0^
F7	mean	1.9310 × 10^3^	2.4859 × 10^3^	3.6656 × 10^3^	3.5548 × 10^3^	1.2055 × 10^4^	3.4556 × 10^3^	1.2102 × 10^4^	3.3425 × 10^3^	5.6263 × 10^3^	3.4906 × 10^3^	3.2834 × 10^3^	2.6046 × 10^3^
	std	1.9336 × 10^2^	3.5410 × 10^2^	1.8671 × 10^2^	1.3194 × 10^2^	6.7245 × 10^2^	1.4942 × 10^2^	4.9136 × 10^2^	2.3969 × 10^2^	3.9818 × 10^2^	1.5693 × 10^2^	1.9165 × 10^2^	8.1318 × 10^1^
F8	mean	1.6581 × 10^3^	1.9770 × 10^3^	2.0341 × 10^3^	2.2749 × 10^3^	3.3048 × 10^3^	2.0662 × 10^3^	3.1968 × 10^3^	1.9594 × 10^3^	2.0318 × 10^3^	2.1104 × 10^3^	2.0049 × 10^3^	1.6702 × 10^3^
	std	7.7515 × 10^1^	2.1795 × 10^2^	3.0489 × 10^1^	8.7933 × 10^1^	1.2395 × 10^2^	8.4005 × 10^1^	9.9289 × 10^1^	2.1150 × 10^2^	1.4369 × 10^2^	5.9972 × 10^1^	6.5791 × 10^1^	4.9479 × 10^1^
F9	mean	6.0135 × 10^4^	5.3976 × 10^4^	5.7983 × 10^4^	5.1146 × 10^4^	1.8895 × 10^5^	5.2315 × 10^4^	1.8141 × 10^5^	3.1862 × 10^4^	4.2118 × 10^4^	4.5908 × 10^4^	3.6853 × 10^4^	3.0554 × 10^4^
	std	5.6835 × 10^3^	1.9578 × 10^4^	5.5381 × 10^3^	3.4822 × 10^3^	1.3739 × 10^4^	7.0990 × 10^3^	1.3169 × 10^4^	7.9575 × 10^3^	4.3950 × 10^3^	5.4467 × 10^3^	3.8356 × 10^3^	1.8353 × 10^3^
F10	mean	1.6112 × 10^4^	2.0124 × 10^4^	3.2166 × 10^4^	2.5587 × 10^4^	3.4078 × 10^4^	2.4590 × 10^4^	3.3289 × 10^4^	1.9880 × 10^4^	1.5535 × 10^4^	2.3895 × 10^4^	2.0994 × 10^4^	1.6721 × 10^4^
	std	1.3922 × 10^3^	4.1579 × 10^3^	4.7389 × 10^2^	9.3680 × 10^2^	7.2730 × 10^2^	1.8571 × 10^3^	8.0971 × 10^2^	3.8254 × 10^3^	1.6013 × 10^3^	2.2183 × 10^3^	1.7852 × 10^3^	6.8311 × 10^2^
F11	mean	5.5082 × 10^3^	1.2994 × 10^5^	2.0793 × 10^5^	1.0047 × 10^5^	4.5115 × 10^5^	1.0318 × 10^5^	4.1373 × 10^5^	2.8720 × 10^4^	8.0193 × 10^4^	4.7338 × 10^4^	6.5927 × 10^4^	1.2607 × 10^4^
	std	7.8387 × 10^2^	3.9820 × 10^4^	3.2477 × 10^4^	2.0703 × 10^4^	7.4983 × 10^4^	2.0204 × 10^4^	5.8962 × 10^4^	1.6852 × 10^4^	2.8207 × 10^4^	8.0111 × 10^3^	1.4250 × 10^4^	1.4629 × 10^3^
F12	mean	1.9315 × 10^8^	2.1943 × 10^9^	6.4257 × 10^9^	1.1456 × 10^11^	2.7801 × 10^11^	5.1881 × 10^9^	2.5099 × 10^11^	3.2564 × 10^10^	7.3962 × 10^8^	1.1653 × 10^10^	2.0530 × 10^10^	4.5209 × 10^8^
	std	6.4261 × 10^7^	8.1147 × 10^8^	7.6621 × 10^8^	2.4020 × 10^10^	2.9384 × 10^10^	1.5215 × 10^9^	2.5618 × 10^10^	4.4735 × 10^10^	2.8725 × 10^8^	3.7579 × 10^9^	9.5426 × 10^9^	7.3371 × 10^7^
F13	mean	2.1439 × 10^5^	1.0409 × 10^8^	2.5152 × 10^4^	2.1896 × 10^10^	6.3415 × 10^10^	1.8314 × 10^8^	5.9511 × 10^10^	2.5207 × 10^9^	5.9130 × 10^4^	5.6564 × 10^8^	2.7126 × 10^9^	5.3201 × 10^5^
	std	9.1403 × 10^4^	1.2228 × 10^8^	5.1916 × 10^3^	6.4544 × 10^9^	1.0951 × 10^10^	1.3609 × 10^8^	1.0263 × 10^10^	6.7668 × 10^9^	2.0832 × 10^4^	3.6837 × 10^8^	2.1081 × 10^9^	9.1890 × 10^4^
F14	mean	1.6929 × 10^6^	1.0114 × 10^7^	4.3416 × 10^7^	1.2523 × 10^6^	2.8907 × 10^8^	1.0307 × 10^7^	2.0241 × 10^8^	2.9805 × 10^6^	1.2923 × 10^6^	5.6790 × 10^6^	9.0924 × 10^6^	1.9009 × 10^6^
	std	7.3655 × 10^5^	8.9530 × 10^6^	1.0916 × 10^7^	7.5832 × 10^5^	1.0941 × 10^8^	3.3532 × 10^6^	6.6580 × 10^7^	5.1294 × 10^6^	5.9305 × 10^5^	2.3965 × 10^6^	5.3195 × 10^6^	3.1197 × 10^5^
F15	mean	3.8409 × 10^4^	2.5386 × 10^7^	4.3138 × 10^3^	4.7115 × 10^9^	2.9896 × 10^10^	2.9369 × 10^7^	2.4322 × 10^10^	1.5839 × 10^9^	9.9209 × 10^7^	1.6142 × 10^7^	4.1581 × 10^8^	9.8767 × 10^4^
	std	1.5797 × 10^4^	4.2384 × 10^7^	1.2470 × 10^3^	2.1347 × 10^9^	6.5440 × 10^9^	2.7073 × 10^7^	4.3845 × 10^9^	3.6645 × 10^9^	5.4314 × 10^8^	1.3577 × 10^7^	6.5657 × 10^8^	1.2761 × 10^4^
F16	mean	5.9023 × 10^3^	8.6327 × 10^3^	1.1152 × 10^4^	1.3122 × 10^4^	2.7906 × 10^4^	1.1317 × 10^4^	2.4361 × 10^4^	9.0805 × 10^3^	7.1024 × 10^3^	1.1695 × 10^4^	9.9139 × 10^3^	7.2233 × 10^3^
	std	7.4294 × 10^2^	1.2257 × 10^3^	2.7198 × 10^2^	2.3579 × 10^3^	3.8252 × 10^3^	1.3929 × 10^3^	3.1986 × 10^3^	2.4697 × 10^3^	8.4138 × 10^2^	1.3186 × 10^3^	1.3788 × 10^3^	4.5936 × 10^2^
F17	mean	5.2135 × 10^3^	8.2577 × 10^3^	7.8288 × 10^3^	2.8828 × 10^4^	1.3481 × 10^7^	8.0902 × 10^3^	9.5828 × 10^6^	2.7447 × 10^4^	6.2810 × 10^3^	7.4052 × 10^3^	1.2082 × 10^4^	5.3440 × 10^3^
	std	4.8549 × 10^2^	1.2441 × 10^3^	1.9766 × 10^2^	4.9208 × 10^4^	1.0991 × 10^7^	1.3704 × 10^3^	7.4423 × 10^6^	7.7080 × 10^4^	6.5333 × 10^2^	1.2525 × 10^3^	1.1775 × 10^4^	2.4747 × 10^2^
F18	mean	2.9703 × 10^6^	1.7444 × 10^7^	7.4587 × 10^7^	2.7474 × 10^6^	5.4712 × 10^8^	1.0270 × 10^7^	3.3874 × 10^8^	3.0589 × 10^6^	2.1624 × 10^6^	8.4424 × 10^6^	7.6992 × 10^6^	2.1353 × 10^6^
	std	1.6847 × 10^6^	1.1891 × 10^7^	2.0434 × 10^7^	2.1554 × 10^6^	2.1677 × 10^8^	3.7278 × 10^6^	1.1721 × 10^8^	9.6891 × 10^6^	1.2873 × 10^6^	5.2353 × 10^6^	4.5529 × 10^6^	4.0130 × 10^5^
F19	mean	1.3983 × 10^5^	3.2923 × 10^7^	7.3914 × 10^3^	6.0907 × 10^9^	3.1967 × 10^10^	5.6972 × 10^7^	2.4464 × 10^10^	1.1938 × 10^9^	3.0443 × 10^5^	3.4562 × 10^7^	2.7124 × 10^8^	2.7859 × 10^6^
	std	1.0726 × 10^5^	2.3692 × 10^7^	5.2099 × 10^3^	3.6484 × 10^9^	7.0427 × 10^9^	3.0206 × 10^7^	6.0497 × 10^9^	3.9181 × 10^9^	2.4613 × 10^5^	3.1005 × 10^7^	4.1897 × 10^8^	1.2052 × 10^6^
F20	mean	5.2029 × 10^3^	6.2214 × 10^3^	7.6732 × 10^3^	5.2612 × 10^3^	8.8850 × 10^3^	6.0953 × 10^3^	8.5595 × 10^3^	5.4585 × 10^3^	5.8445 × 10^3^	5.5406 × 10^3^	6.0480 × 10^3^	4.8782 × 10^3^
	std	4.2759 × 10^2^	6.3022 × 10^2^	2.4911 × 10^2^	2.7328 × 10^2^	2.4668 × 10^2^	6.8116 × 10^2^	3.6335 × 10^2^	6.4669 × 10^2^	6.3348 × 10^2^	4.7647 × 10^2^	5.8050 × 10^2^	2.0478 × 10^2^
F21	mean	3.5566 × 10^3^	3.8161 × 10^3^	3.5552 × 10^3^	3.9587 × 10^3^	5.0584 × 10^3^	3.9986 × 10^3^	4.8757 × 10^3^	4.0417 × 10^3^	4.0718 × 10^3^	3.8261 × 10^3^	3.6868 × 10^3^	3.3045 × 10^3^
	std	1.6298 × 10^2^	1.5814 × 10^2^	1.9908 × 10^1^	1.1317 × 10^2^	1.3765 × 10^2^	1.7688 × 10^2^	1.2379 × 10^2^	2.4963 × 10^2^	2.1573 × 10^2^	1.5736 × 10^2^	1.5412 × 10^2^	6.5374 × 10^1^
F22	mean	1.9438 × 10^4^	2.3606 × 10^4^	3.4357 × 10^4^	2.9350 × 10^4^	3.6714 × 10^4^	2.7450 × 10^4^	3.5858 × 10^4^	2.3450 × 10^4^	1.9149 × 10^4^	2.7190 × 10^4^	2.4304 × 10^4^	2.0667 × 10^4^
	std	1.4741 × 10^3^	4.5183 × 10^3^	5.9876 × 10^2^	7.5912 × 10^2^	6.7439 × 10^2^	1.6484 × 10^3^	6.0757 × 10^2^	4.3770 × 10^3^	1.7701 × 10^3^	1.2764 × 10^3^	1.6426 × 10^3^	7.0815 × 10^2^
F23	mean	4.8375 × 10^3^	4.4524 × 10^3^	3.9615 × 10^3^	4.6464 × 10^3^	6.8251 × 10^3^	4.8022 × 10^3^	6.1770 × 10^3^	4.9967 × 10^3^	4.8377 × 10^3^	4.6312 × 10^3^	4.3476 × 10^3^	3.9001 × 10^3^
	std	3.4874 × 10^2^	1.7784 × 10^2^	2.8351 × 10^1^	1.7691 × 10^2^	3.4460 × 10^2^	2.0527 × 10^2^	2.1078 × 10^2^	3.0879 × 10^2^	1.9663 × 10^2^	2.2894 × 10^2^	1.7691 × 10^2^	7.6276 × 10^1^
F24	mean	5.4915 × 10^3^	5.5158 × 10^3^	4.4254 × 10^3^	6.0469 × 10^3^	1.1242 × 10^4^	6.0808 × 10^3^	9.6470 × 10^3^	6.5956 × 10^3^	6.4250 × 10^3^	5.5311 × 10^3^	5.4512 × 10^3^	4.6226 × 10^3^
	std	4.7362 × 10^2^	2.8658 × 10^2^	2.7806 × 10^1^	2.8678 × 10^2^	7.9258 × 10^2^	3.4380 × 10^2^	6.6463 × 10^2^	6.5386 × 10^2^	3.7405 × 10^2^	3.5290 × 10^2^	3.7562 × 10^2^	6.4913 × 10^1^
F25	mean	3.3535 × 10^3^	8.8468 × 10^3^	1.3265 × 10^4^	2.2048 × 10^4^	1.0759 × 10^5^	5.6565 × 10^3^	9.9680 × 10^4^	9.9253 × 10^3^	3.9953 × 10^3^	7.4599 × 10^3^	8.1929 × 10^3^	3.7610 × 10^3^
	std	4.7772 × 10^1^	5.2477 × 10^3^	8.7086 × 10^2^	2.0174 × 10^3^	1.3457 × 10^4^	4.0243 × 10^2^	1.4290 × 10^4^	5.5418 × 10^3^	2.6511 × 10^2^	6.6584 × 10^2^	1.4645 × 10^3^	3.5168 × 10^1^
F26	mean	1.7600 × 10^4^	2.4786 × 10^4^	1.7903 × 10^4^	4.2072 × 10^4^	7.1745 × 10^4^	2.9723 × 10^4^	6.3946 × 10^4^	3.2939 × 10^4^	3.1013 × 10^4^	2.8991 × 10^4^	3.0282 × 10^4^	1.3552 × 10^4^
	std	7.5967 × 10^3^	3.1601 × 10^3^	4.3060 × 10^2^	2.9494 × 10^3^	7.6872 × 10^3^	4.7466 × 10^3^	5.0715 × 10^3^	6.2733 × 10^3^	2.5840 × 10^3^	3.4741 × 10^3^	2.0934 × 10^3^	5.1113 × 10^3^
F27	mean	3.4709 × 10^3^	4.2447 × 10^3^	4.5080 × 10^3^	5.6710 × 10^3^	1.3122 × 10^4^	4.6439 × 10^3^	1.1341 × 10^4^	5.5933 × 10^3^	5.5228 × 10^3^	4.8841 × 10^3^	5.2115 × 10^3^	4.1508 × 10^3^
	std	2.0127 × 10^2^	2.8770 × 10^2^	1.0691 × 10^2^	4.3914 × 10^2^	1.2431 × 10^3^	2.9640 × 10^2^	1.3540 × 10^3^	1.0501 × 10^3^	5.4416 × 10^2^	3.7166 × 10^2^	4.9903 × 10^2^	1.1908 × 10^2^
F28	mean	3.4068 × 10^3^	1.8846 × 10^4^	1.5219 × 10^4^	2.5772 × 10^4^	6.0657 × 10^4^	7.0005 × 10^3^	5.5754 × 10^4^	1.5308 × 10^4^	4.1726 × 10^3^	9.3222 × 10^3^	1.0817 × 10^4^	3.8440 × 10^3^
	std	5.9616 × 10^1^	6.5191 × 10^3^	8.9659 × 10^2^	2.4713 × 10^3^	4.8545 × 10^3^	8.0981 × 10^2^	4.6421 × 10^3^	8.7921 × 10^3^	4.2315 × 10^2^	1.1253 × 10^3^	1.7695 × 10^3^	3.9568 × 10^1^
F29	mean	8.3723 × 10^3^	1.0313 × 10^4^	1.0708 × 10^4^	2.5008 × 10^4^	2.4034 × 10^6^	1.4682 × 10^4^	1.3342 × 10^6^	1.5583 × 10^4^	1.0435 × 10^4^	1.3225 × 10^4^	1.2871 × 10^4^	9.5424 × 10^3^
	std	6.1003 × 10^2^	1.6888 × 10^3^	3.7774 × 10^2^	1.4571 × 10^4^	2.3469 × 10^6^	1.8804 × 10^3^	9.0266 × 10^5^	1.1953 × 10^4^	1.1081 × 10^3^	1.3959 × 10^3^	1.5185 × 10^3^	4.2615 × 10^2^
F30	mean	6.3701 × 10^6^	9.7467 × 10^7^	1.0439 × 10^7^	1.4970 × 10^10^	5.0011 × 10^10^	7.4598 × 10^8^	4.1053 × 10^10^	2.4285 × 10^9^	1.1978 × 10^7^	8.2198 × 10^8^	1.4441 × 10^9^	9.0562 × 10^7^
	std	3.2492 × 10^6^	6.1779 × 10^7^	4.1847 × 10^6^	7.4639 × 10^9^	9.7578 × 10^9^	3.4036 × 10^8^	7.5905 × 10^9^	5.5095 × 10^9^	5.9905 × 10^6^	3.2161 × 10^8^	1.1092 × 10^9^	1.5677 × 10^7^

**Table 5:** *p*-values for various algorithms on the CEC 2017 (dim = 30).

Item	PSO	DBO	DMO	IAO	MGO	PO	PLO	BKA	CPSOGSA	HPHHO	SCSO
F1	3.3384 × 10^−11^	2.7548 × 10^−3^	1.2967 × 10^−1^	3.0199 × 10^−11^	3.0199 × 10^−11^	3.0199 × 10^−11^	3.0199 × 10^−11^	3.0199 × 10^−11^	3.0199 × 10^−11^	3.0199 × 10^−11^	3.0199 × 10^−11^
F2	3.0199 × 10^−11^	3.0199 × 10^−11^	3.0199 × 10^−11^	3.0199 × 10^−11^	3.0199 × 10^−11^	3.0199 × 10^−11^	3.0199 × 10^−11^	1.6132 × 10^−10^	3.0199 × 10^−11^	1.2118 × 10^−12^	3.0199 × 10^−11^
F3	2.1959 × 10^−7^	3.0199 × 10^−11^	3.0199 × 10^−11^	3.0199 × 10^−11^	3.0199 × 10^−11^	3.0199 × 10^−11^	3.0199 × 10^−11^	1.1199 × 10^−1^	3.0199 × 10^−11^	3.0199 × 10^−11^	3.0199 × 10^−11^
F4	2.7719 × 10^−1^	6.1210 × 10^−10^	4.5043 × 10^−11^	3.0199 × 10^−11^	3.0199 × 10^−11^	3.0199 × 10^−11^	3.0199 × 10^−11^	4.5726 × 10^−9^	4.4272 × 10^−3^	3.0199 × 10^−11^	3.0199 × 10^−11^
F5	8.8829 × 10^−6^	5.4941 × 10^−11^	3.0199 × 10^−11^	8.9934 × 10^−11^	3.0199 × 10^−11^	3.0199 × 10^−11^	3.0199 × 10^−11^	3.0199 × 10^−11^	3.0199 × 10^−11^	3.0199 × 10^−11^	3.0199 × 10^−11^
F6	9.0307 × 10^−4^	3.3285 × 10^−1^	3.0199 × 10^−11^	7.0881 × 10^−8^	3.0199 × 10^−11^	3.0199 × 10^−11^	3.0199 × 10^−11^	3.0199 × 10^−11^	3.0199 × 10^−11^	2.3715 × 10^−10^	1.4643 × 10^−10^
F7	1.8567 × 10^−9^	2.9047 × 10^−1^	1.0937 × 10^−10^	3.0199 × 10^−11^	3.0199 × 10^−11^	3.0199 × 10^−11^	3.0199 × 10^−11^	3.0199 × 10^−11^	3.0199 × 10^−11^	3.0199 × 10^−11^	3.0199 × 10^−11^
F8	6.3772 × 10^−3^	2.9215 × 10^−9^	3.0199 × 10^−11^	3.0199 × 10^−11^	3.0199 × 10^−11^	3.0199 × 10^−11^	3.0199 × 10^−11^	1.2023 × 10^−8^	3.0199 × 10^−11^	1.4643 × 10^−10^	3.0199 × 10^−11^
F9	9.5139 × 10^−6^	2.1959 × 10^−7^	3.0199 × 10^−11^	1.0105 × 10^−8^	3.0199 × 10^−11^	3.0199 × 10^−11^	3.0199 × 10^−11^	5.4941 × 10^−11^	3.0199 × 10^−11^	3.0199 × 10^−11^	3.0199 × 10^−11^
F10	1.1711 × 10^−2^	1.3853 × 10^−6^	3.0199 × 10^−11^	3.0199 × 10^−11^	3.0199 × 10^−11^	5.5727 × 10^−10^	3.0199 × 10^−11^	1.5292 × 10^−5^	2.1959 × 10^−7^	1.1077 × 10^−6^	1.6947 × 10^−9^
F11	9.3341 × 10^−2^	3.0199 × 10^−11^	4.5043 × 10^−11^	3.0199 × 10^−11^	3.0199 × 10^−11^	3.0199 × 10^−11^	3.0199 × 10^−11^	2.3768 × 10^−7^	4.2175 × 10^−4^	3.0199 × 10^−11^	3.0199 × 10^−11^
F12	2.2273 × 10^−9^	1.0407 × 10^−4^	7.3803 × 10^−10^	3.0199 × 10^−11^	3.0199 × 10^−11^	3.0199 × 10^−11^	3.0199 × 10^−11^	5.2640 × 10^−4^	6.5277 × 10^−8^	3.0199 × 10^−11^	3.0199 × 10^−11^
F13	2.3168 × 10^−6^	1.7294 × 10^−7^	1.0702 × 10^−9^	7.2884 × 10^−3^	3.0199 × 10^−11^	3.0199 × 10^−11^	3.0199 × 10^−11^	2.0338 × 10^−9^	4.1191 × 10^−1^	3.0199 × 10^−11^	2.7829 × 10^−7^
F14	6.5671 × 10^−2^	2.4386 × 10^−9^	3.0199 × 10^−11^	3.0199 × 10^−11^	3.0199 × 10^−11^	3.0199 × 10^−11^	3.0199 × 10^−11^	2.3715 × 10^−10^	2.6243 × 10^−3^	5.0723 × 10^−10^	8.4848 × 10^−9^
F15	4.9818 × 10^−4^	1.0105 × 10^−8^	3.1967 × 10^−9^	6.0658 × 10^−11^	3.0199 × 10^−11^	4.5043 × 10^−11^	3.0199 × 10^−11^	7.5991 × 10^−7^	2.3985 × 10^−1^	3.4742 × 10^−10^	3.0199 × 10^−11^
F16	4.8413 × 10^−2^	4.7445 × 10^−6^	6.0658 × 10^−11^	4.4642 × 10^−1^	3.0199 × 10^−11^	8.1527 × 10^−11^	3.0199 × 10^−11^	1.4918 × 10^−6^	9.2603 × 10^−9^	1.3367 × 10^−5^	3.1589 × 10^−10^
F17	2.1540 × 10^−6^	5.4941 × 10^−11^	5.5727 × 10^−10^	1.0547 × 10^−1^	3.0199 × 10^−11^	2.3715 × 10^−10^	3.0199 × 10^−11^	9.2603 × 10^−9^	3.0199 × 10^−11^	1.7294 × 10^−7^	8.1014 × 10^−10^
F18	1.5178 × 10^−3^	3.2555 × 10^−7^	3.0199 × 10^−11^	3.0199 × 10^−11^	3.0199 × 10^−11^	2.1544 × 10^−10^	3.0199 × 10^−11^	5.1857 × 10^−7^	8.5000 × 10^−2^	2.9215 × 10^−9^	1.1567 × 10^−7^
F19	2.4386 × 10^−9^	7.0617 × 10^−1^	1.8567 × 10^−9^	3.0199 × 10^−11^	3.0199 × 10^−11^	1.0937 × 10^−10^	3.0199 × 10^−11^	9.9410 × 10^−1^	2.0152 × 10^−8^	2.0523 × 10^−3^	3.4742 × 10^−10^
F20	3.5708 × 10^−6^	2.9215 × 10^−9^	3.0199 × 10^−11^	5.8282 × 10^−3^	3.0199 × 10^−11^	4.1997 × 10^−10^	3.0199 × 10^−11^	7.2208 × 10^−6^	1.2870 × 10^−9^	1.5964 × 10^−7^	1.2023 × 10^−8^
F21	3.6459 × 10^−8^	3.0199 × 10^−11^	3.0199 × 10^−11^	9.5332 × 10^−7^	3.0199 × 10^−11^	3.0199 × 10^−11^	3.0199 × 10^−11^	2.6695 × 10^−9^	3.0199 × 10^−11^	3.0199 × 10^−11^	1.7769 × 10^−10^
F22	1.0000 × 10^0^	7.3803 × 10^−10^	3.0199 × 10^−11^	3.0199 × 10^−11^	3.0199 × 10^−11^	3.0199 × 10^−11^	3.0199 × 10^−11^	3.0199 × 10^−11^	3.9881 × 10^−4^	3.0199 × 10^−11^	3.0199 × 10^−11^
F23	3.0199 × 10^−11^	1.9568 × 10^−10^	3.0199 × 10^−11^	3.0199 × 10^−11^	3.0199 × 10^−11^	3.4742 × 10^−10^	3.0199 × 10^−11^	3.0199 × 10^−11^	3.0199 × 10^−11^	3.0199 × 10^−11^	3.0199 × 10^−11^
F24	3.0199 × 10^−11^	4.5043 × 10^−11^	3.0199 × 10^−11^	3.0199 × 10^−11^	3.0199 × 10^−11^	3.0199 × 10^−11^	3.0199 × 10^−11^	3.0199 × 10^−11^	3.0199 × 10^−11^	3.0199 × 10^−11^	3.1589 × 10^−10^
F25	5.0912 × 10^−6^	6.0459 × 10^−7^	6.5261 × 10^−7^	3.0199 × 10^−11^	3.0199 × 10^−11^	3.0199 × 10^−11^	3.0199 × 10^−11^	2.6099 × 10^−10^	4.3531 × 10^−5^	3.0199 × 10^−11^	3.0199 × 10^−11^
F26	4.1178 × 10^−6^	3.0199 × 10^−11^	3.0199 × 10^−11^	3.0199 × 10^−11^	3.0199 × 10^−11^	3.0199 × 10^−11^	3.0199 × 10^−11^	3.0199 × 10^−11^	3.0199 × 10^−11^	3.0199 × 10^−11^	3.0199 × 10^−11^
F27	9.9410 × 10^−1^	5.7460 × 10^−2^	6.6955 × 10^−11^	1.2362 × 10^−3^	3.0199 × 10^−11^	3.0199 × 10^−11^	3.0199 × 10^−11^	8.8910 × 10^−10^	3.4742 × 10^−10^	4.1127 × 10^−7^	1.0702 × 10^−9^
F28	6.2040 × 10^−1^	3.6897 × 10^−11^	3.0199 × 10^−11^	3.0199 × 10^−11^	3.0199 × 10^−11^	3.0199 × 10^−11^	3.0199 × 10^−11^	1.7769 × 10^−10^	6.7362 × 10^−6^	3.0199 × 10^−11^	3.0199 × 10^−11^
F29	2.8129 × 10^−2^	3.6439 × 10^−2^	2.8314 × 10^−8^	9.2344 × 10^−1^	3.0199 × 10^−11^	8.1527 × 10^−11^	3.0199 × 10^−11^	1.5964 × 10^−7^	6.5261 × 10^−7^	1.2362 × 10^−3^	9.2603 × 10^−9^
F30	3.0199 × 10^−11^	2.2360 × 10^−2^	7.3891 × 10^−11^	4.9752 × 10^−11^	3.0199 × 10^−11^	3.0199 × 10^−11^	3.0199 × 10^−11^	2.1702 × 10^−1^	4.0772 × 10^−11^	1.6947 × 10^−9^	3.0199 × 10^−11^

**Table 6:** *p*-values for various algorithms on the CEC 2017 (dim = 50).

Item	PSO	DBO	DMO	IAO	MGO	PO	PLO	BKA	CPSOGSA	HPHHO	SCSO
F1	1.2870 × 10^−9^	3.0199 × 10^−11^	3.0199 × 10^−11^	3.0199 × 10^−11^	3.0199 × 10^−11^	3.0199 × 10^−11^	3.0199 × 10^−11^	3.0199 × 10^−11^	9.3519 × 10^−1^	3.0199 × 10^−11^	3.0199 × 10^−11^
F2	3.0199 × 10^−11^	3.0199 × 10^−11^	3.0199 × 10^−11^	3.0199 × 10^−11^	3.0199 × 10^−11^	3.0199 × 10^−11^	3.0199 × 10^−11^	3.0199 × 10^−11^	3.0199 × 10^−11^	1.2118 × 10^−12^	3.0199 × 10^−11^
F3	3.0103 × 10^−7^	3.0199 × 10^−11^	3.0199 × 10^−11^	3.0199 × 10^−11^	3.0199 × 10^−11^	3.0199 × 10^−11^	3.0199 × 10^−11^	6.5204 × 10^−1^	3.0199 × 10^−11^	2.1544 × 10^−10^	3.0199 × 10^−11^
F4	9.0688 × 10^−3^	3.0199 × 10^−11^	3.0199 × 10^−11^	3.0199 × 10^−11^	3.0199 × 10^−11^	3.0199 × 10^−11^	3.0199 × 10^−11^	3.0199 × 10^−11^	1.1937 × 10^−6^	3.0199 × 10^−11^	3.0199 × 10^−11^
F5	2.2823 × 10^−1^	3.0199 × 10^−11^	3.0199 × 10^−11^	3.0199 × 10^−11^	3.0199 × 10^−11^	3.0199 × 10^−11^	3.0199 × 10^−11^	3.0199 × 10^−11^	3.0199 × 10^−11^	3.0199 × 10^−11^	3.0199 × 10^−11^
F6	5.5699 × 10^−3^	3.5547 × 10^−1^	3.0199 × 10^−11^	5.9673 × 10^−9^	3.0199 × 10^−11^	3.0199 × 10^−11^	3.0199 × 10^−11^	4.6159 × 10^−10^	6.0658 × 10^−11^	3.0199 × 10^−11^	3.6897 × 10^−11^
F7	6.5277 × 10^−8^	3.7904 × 10^−1^	2.6806 × 10^−4^	3.0199 × 10^−11^	3.0199 × 10^−11^	3.0199 × 10^−11^	3.0199 × 10^−11^	3.0199 × 10^−11^	3.0199 × 10^−11^	3.0199 × 10^−11^	3.0199 × 10^−11^
F8	6.3088 × 10^−1^	3.1589 × 10^−10^	3.0199 × 10^−11^	3.0199 × 10^−11^	3.0199 × 10^−11^	3.0199 × 10^−11^	3.0199 × 10^−11^	3.0199 × 10^−11^	3.0199 × 10^−11^	3.0199 × 10^−11^	3.0199 × 10^−11^
F9	3.0199 × 10^−11^	3.3386 × 10^−3^	3.4742 × 10^−10^	1.0937 × 10^−10^	3.0199 × 10^−11^	3.0199 × 10^−11^	3.0199 × 10^−11^	6.1210 × 10^−10^	6.0658 × 10^−11^	3.0199 × 10^−11^	2.6099 × 10^−10^
F10	1.1882 × 10^−1^	7.5991 × 10^−7^	3.0199 × 10^−11^	3.0199 × 10^−11^	3.0199 × 10^−11^	3.0199 × 10^−11^	3.0199 × 10^−11^	1.0666 × 10^−7^	1.7290 × 10^−6^	4.1997 × 10^−10^	3.0199 × 10^−11^
F11	3.6897 × 10^−11^	3.0199 × 10^−11^	3.0199 × 10^−11^	3.0199 × 10^−11^	3.0199 × 10^−11^	3.0199 × 10^−11^	3.0199 × 10^−11^	1.3111 × 10^−8^	1.0277 × 10^−6^	3.0199 × 10^−11^	3.0199 × 10^−11^
F12	2.8716 × 10^−10^	6.6955 × 10^−11^	3.0199 × 10^−11^	3.0199 × 10^−11^	3.0199 × 10^−11^	3.0199 × 10^−11^	3.0199 × 10^−11^	5.0912 × 10^−6^	1.0035 × 10^−3^	3.0199 × 10^−11^	3.1589 × 10^−10^
F13	3.0199 × 10^−11^	2.0283 × 10^−7^	3.0199 × 10^−11^	3.0199 × 10^−11^	3.0199 × 10^−11^	3.0199 × 10^−11^	3.0199 × 10^−11^	3.0199 × 10^−11^	7.1186 × 10^−9^	3.0199 × 10^−11^	3.0199 × 10^−11^
F14	4.2259 × 10^−3^	7.7387 × 10^−6^	3.0199 × 10^−11^	3.0199 × 10^−11^	3.0199 × 10^−11^	3.0199 × 10^−11^	3.0199 × 10^−11^	2.4913 × 10^−6^	5.6922 × 10^−1^	2.2273 × 10^−9^	2.0152 × 10^−8^
F15	9.7555 × 10^−10^	5.5727 × 10^−10^	4.9752 × 10^−11^	1.2597 × 10^−1^	3.0199 × 10^−11^	3.0199 × 10^−11^	3.0199 × 10^−11^	2.0152 × 10^−8^	6.5671 × 10^−2^	3.0199 × 10^−11^	3.0199 × 10^−11^
F16	9.1171 × 10^−1^	3.1589 × 10^−10^	3.0199 × 10^−11^	5.9673 × 10^−9^	3.0199 × 10^−11^	6.1210 × 10^−10^	3.0199 × 10^−11^	1.6351 × 10^−5^	1.7290 × 10^−6^	3.0811 × 10^−8^	1.3289 × 10^−10^
F17	7.7272 × 10^−2^	5.0723 × 10^−10^	3.0199 × 10^−11^	3.5137 × 10^−2^	3.0199 × 10^−11^	4.0772 × 10^−11^	3.0199 × 10^−11^	2.8790 × 10^−6^	1.6132 × 10^−10^	8.4848 × 10^−9^	3.8249 × 10^−9^
F18	3.0339 × 10^−3^	1.4733 × 10^−7^	3.0199 × 10^−11^	3.0199 × 10^−11^	3.0199 × 10^−11^	3.0199 × 10^−11^	3.0199 × 10^−11^	9.4683 × 10^−3^	5.2978 × 10^−1^	4.6159 × 10^−10^	5.4617 × 10^−9^
F19	3.3384 × 10^−11^	4.0595 × 10^−2^	3.0199 × 10^−11^	1.8368 × 10^−2^	3.0199 × 10^−11^	3.4742 × 10^−10^	3.0199 × 10^−11^	9.0688 × 10^−3^	3.6897 × 10^−11^	1.1536 × 10^−1^	1.1737 × 10^−9^
F20	7.6171 × 10^−3^	4.5726 × 10^−9^	3.0199 × 10^−11^	1.2967 × 10^−1^	3.0199 × 10^−11^	4.1997 × 10^−10^	3.0199 × 10^−11^	6.7362 × 10^−6^	7.1186 × 10^−9^	7.2951 × 10^−4^	2.6695 × 10^−9^
F21	1.1937 × 10^−6^	3.0199 × 10^−11^	3.0199 × 10^−11^	3.0199 × 10^−11^	3.0199 × 10^−11^	3.0199 × 10^−11^	3.0199 × 10^−11^	3.0199 × 10^−11^	3.0199 × 10^−11^	3.0199 × 10^−11^	3.0199 × 10^−11^
F22	4.3764 × 10^−1^	8.1527 × 10^−11^	3.0199 × 10^−11^	8.8910 × 10^−10^	3.0199 × 10^−11^	3.0199 × 10^−11^	3.0199 × 10^−11^	1.5292 × 10^−5^	8.2919 × 10^−6^	3.0199 × 10^−11^	2.8716 × 10^−10^
F23	2.1544 × 10^−10^	6.6955 × 10^−11^	3.0199 × 10^−11^	3.0199 × 10^−11^	3.0199 × 10^−11^	3.0199 × 10^−11^	3.0199 × 10^−11^	3.0199 × 10^−11^	3.0199 × 10^−11^	3.0199 × 10^−11^	4.6159 × 10^−10^
F24	3.0199 × 10^−11^	3.0199 × 10^−11^	3.0199 × 10^−11^	3.0199 × 10^−11^	3.0199 × 10^−11^	3.0199 × 10^−11^	3.0199 × 10^−11^	3.0199 × 10^−11^	3.0199 × 10^−11^	3.0199 × 10^−11^	5.4941 × 10^−11^
F25	7.3891 × 10^−11^	2.6099 × 10^−10^	3.0199 × 10^−11^	3.0199 × 10^−11^	3.0199 × 10^−11^	3.0199 × 10^−11^	3.0199 × 10^−11^	3.0199 × 10^−11^	5.9428 × 10^−2^	3.0199 × 10^−11^	3.0199 × 10^−11^
F26	1.9527 × 10^−3^	3.0199 × 10^−11^	3.0199 × 10^−11^	3.0199 × 10^−11^	3.0199 × 10^−11^	3.0199 × 10^−11^	3.0199 × 10^−11^	3.0199 × 10^−11^	3.0199 × 10^−11^	3.0199 × 10^−11^	3.0199 × 10^−11^
F27	9.7052 × 10^−1^	3.3874 × 10^−2^	2.8716 × 10^−10^	1.8567 × 10^−9^	3.0199 × 10^−11^	4.6856 × 10^−8^	3.0199 × 10^−11^	3.0199 × 10^−11^	3.0199 × 10^−11^	1.0666 × 10^−7^	8.1527 × 10^−11^
F28	2.2273 × 10^−9^	7.3803 × 10^−10^	3.0199 × 10^−11^	3.0199 × 10^−11^	3.0199 × 10^−11^	3.0199 × 10^−11^	3.0199 × 10^−11^	3.0199 × 10^−11^	3.5923 × 10^−5^	3.0199 × 10^−11^	3.0199 × 10^−11^
F29	7.9590 × 10^−3^	1.2597 × 10^−1^	1.8731 × 10^−7^	8.8411 × 10^−7^	3.0199 × 10^−11^	4.0772 × 10^−11^	3.0199 × 10^−11^	1.0277 × 10^−6^	2.3715 × 10^−10^	1.4110 × 10^−9^	1.1567 × 10^−7^
F30	3.0199 × 10^−11^	9.8329 × 10^−8^	3.0199 × 10^−11^	4.6756 × 10^−2^	3.0199 × 10^−11^	8.1527 × 10^−11^	3.0199 × 10^−11^	3.6459 × 10^−8^	1.1567 × 10^−7^	7.3803 × 10^−10^	4.5726 × 10^−9^

**Table 7:** *p*-values for various algorithms on the CEC 2017 (dim = 100).

Item	PSO	DBO	DMO	IAO	MGO	PO	PLO	BKA	CPSOGSA	HPHHO	SCSO
F1	3.0199 × 10^−11^	3.0199 × 10^−11^	3.0199 × 10^−11^	3.0199 × 10^−11^	3.0199 × 10^−11^	3.0199 × 10^−11^	3.0199 × 10^−11^	3.0199 × 10^−11^	1.6947 × 10^−9^	3.0199 × 10^−11^	3.0199 × 10^−11^
F2	3.0199 × 10^−11^	3.0199 × 10^−11^	3.0199 × 10^−11^	3.0199 × 10^−11^	3.0199 × 10^−11^	3.0199 × 10^−11^	3.0199 × 10^−11^	3.0199 × 10^−11^	3.0199 × 10^−11^	1.2118 × 10^−12^	3.0199 × 10^−11^
F3	1.9568 × 10^−10^	3.0199 × 10^−11^	3.0199 × 10^−11^	5.1857 × 10^−7^	3.0199 × 10^−11^	3.0199 × 10^−11^	3.0199 × 10^−11^	1.4918 × 10^−6^	3.0199 × 10^−11^	1.3703 × 10^−3^	3.6459 × 10^−8^
F4	3.0199 × 10^−11^	3.0199 × 10^−11^	3.0199 × 10^−11^	3.0199 × 10^−11^	3.0199 × 10^−11^	3.0199 × 10^−11^	3.0199 × 10^−11^	3.0199 × 10^−11^	3.0199 × 10^−11^	3.0199 × 10^−11^	3.0199 × 10^−11^
F5	5.9969 × 10^−1^	3.4971 × 10^−9^	3.0199 × 10^−11^	3.0199 × 10^−11^	3.0199 × 10^−11^	3.0199 × 10^−11^	3.0199 × 10^−11^	3.0199 × 10^−11^	3.0199 × 10^−11^	3.0199 × 10^−11^	3.0199 × 10^−11^
F6	1.5292 × 10^−5^	2.0523 × 10^−3^	3.0199 × 10^−11^	3.0199 × 10^−11^	3.0199 × 10^−11^	3.0199 × 10^−11^	3.0199 × 10^−11^	8.8411 × 10^−7^	1.6980 × 10^−8^	3.0199 × 10^−11^	3.0199 × 10^−11^
F7	3.3384 × 10^−11^	1.4412 × 10^−2^	3.0199 × 10^−11^	3.0199 × 10^−11^	3.0199 × 10^−11^	3.0199 × 10^−11^	3.0199 × 10^−11^	3.0199 × 10^−11^	3.0199 × 10^−11^	3.0199 × 10^−11^	3.0199 × 10^−11^
F8	2.5805 × 10^−1^	3.8249 × 10^−9^	3.0199 × 10^−11^	3.0199 × 10^−11^	3.0199 × 10^−11^	3.0199 × 10^−11^	3.0199 × 10^−11^	3.0199 × 10^−11^	1.2057 × 10^−10^	3.0199 × 10^−11^	3.0199 × 10^−11^
F9	3.0199 × 10^−11^	1.3367 × 10^−5^	3.0199 × 10^−11^	3.0199 × 10^−11^	3.0199 × 10^−11^	3.0199 × 10^−11^	3.0199 × 10^−11^	5.8945 × 10^−1^	3.0199 × 10^−11^	3.0199 × 10^−11^	5.9673 × 10^−9^
F10	6.5671 × 10^−2^	3.0103 × 10^−7^	3.0199 × 10^−11^	3.0199 × 10^−11^	3.0199 × 10^−11^	3.0199 × 10^−11^	3.0199 × 10^−11^	2.9215 × 10^−9^	1.5969 × 10^−3^	3.0199 × 10^−11^	3.0199 × 10^−11^
F11	3.0199 × 10^−11^	3.0199 × 10^−11^	3.0199 × 10^−11^	3.0199 × 10^−11^	3.0199 × 10^−11^	3.0199 × 10^−11^	3.0199 × 10^−11^	3.0199 × 10^−11^	3.0199 × 10^−11^	3.0199 × 10^−11^	3.0199 × 10^−11^
F12	4.9752 × 10^−11^	3.0199 × 10^−11^	3.0199 × 10^−11^	3.0199 × 10^−11^	3.0199 × 10^−11^	3.0199 × 10^−11^	3.0199 × 10^−11^	3.0199 × 10^−11^	6.7362 × 10^−6^	3.0199 × 10^−11^	3.0199 × 10^−11^
F13	8.9934 × 10^−11^	4.0772 × 10^−11^	3.0199 × 10^−11^	3.0199 × 10^−11^	3.0199 × 10^−11^	3.0199 × 10^−11^	3.0199 × 10^−11^	3.0199 × 10^−11^	3.0199 × 10^−11^	3.0199 × 10^−11^	3.0199 × 10^−11^
F14	1.3832 × 10^−2^	2.5721 × 10^−7^	3.0199 × 10^−11^	7.1988 × 10^−5^	3.0199 × 10^−11^	3.0199 × 10^−11^	3.0199 × 10^−11^	1.7836 × 10^−4^	6.7362 × 10^−6^	4.5043 × 10^−11^	4.6159 × 10^−10^
F15	3.6897 × 10^−11^	1.2057 × 10^−10^	3.0199 × 10^−11^	3.0199 × 10^−11^	3.0199 × 10^−11^	3.0199 × 10^−11^	3.0199 × 10^−11^	3.0199 × 10^−11^	1.5465 × 10^−9^	3.0199 × 10^−11^	3.0199 × 10^−11^
F16	1.1023 × 10^−8^	4.6856 × 10^−8^	3.0199 × 10^−11^	3.0199 × 10^−11^	3.0199 × 10^−11^	3.0199 × 10^−11^	3.0199 × 10^−11^	5.1857 × 10^−7^	5.9428 × 10^−2^	3.0199 × 10^−11^	3.0199 × 10^−11^
F17	1.5798 × 10^−1^	3.0199 × 10^−11^	3.0199 × 10^−11^	3.0199 × 10^−11^	3.0199 × 10^−11^	3.0199 × 10^−11^	3.0199 × 10^−11^	3.8202 × 10^−10^	3.2555 × 10^−7^	5.0723 × 10^−10^	5.5727 × 10^−10^
F18	1.4128 × 10^−1^	3.0199 × 10^−11^	3.0199 × 10^−11^	7.7312 × 10^−1^	3.0199 × 10^−11^	3.0199 × 10^−11^	3.0199 × 10^−11^	1.0188 × 10^−5^	2.7071 × 10^−1^	3.0199 × 10^−11^	2.9215 × 10^−9^
F19	3.0199 × 10^−11^	2.6695 × 10^−9^	3.0199 × 10^−11^	3.0199 × 10^−11^	3.0199 × 10^−11^	3.0199 × 10^−11^	3.0199 × 10^−11^	7.7725 × 10^−9^	5.4941 × 10^−11^	3.0199 × 10^−11^	3.0199 × 10^−11^
F20	7.9590 × 10^−3^	5.5727 × 10^−10^	3.0199 × 10^−11^	3.8053 × 10^−7^	3.0199 × 10^−11^	3.1589 × 10^−10^	3.0199 × 10^−11^	2.0023 × 10^−6^	3.9648 × 10^−8^	1.1567 × 10^−7^	6.6955 × 10^−11^
F21	7.7725 × 10^−9^	3.0199 × 10^−11^	3.0199 × 10^−11^	3.0199 × 10^−11^	3.0199 × 10^−11^	3.0199 × 10^−11^	3.0199 × 10^−11^	3.0199 × 10^−11^	3.0199 × 10^−11^	3.0199 × 10^−11^	3.0199 × 10^−11^
F22	1.7836 × 10^−4^	1.1711 × 10^−2^	3.0199 × 10^−11^	3.0199 × 10^−11^	3.0199 × 10^−11^	3.0199 × 10^−11^	3.0199 × 10^−11^	1.7836 × 10^−4^	1.3017 × 10^−3^	3.0199 × 10^−11^	2.6099 × 10^−10^
F23	3.0199 × 10^−11^	3.0199 × 10^−11^	3.3679 × 10^−4^	3.0199 × 10^−11^	3.0199 × 10^−11^	3.0199 × 10^−11^	3.0199 × 10^−11^	3.0199 × 10^−11^	3.0199 × 10^−11^	3.0199 × 10^−11^	3.0199 × 10^−11^
F24	1.3289 × 10^−10^	3.0199 × 10^−11^	1.7769 × 10^−10^	3.0199 × 10^−11^	3.0199 × 10^−11^	3.0199 × 10^−11^	3.0199 × 10^−11^	3.0199 × 10^−11^	3.0199 × 10^−11^	3.0199 × 10^−11^	3.0199 × 10^−11^
F25	3.0199 × 10^−11^	4.1825 × 10^−9^	3.0199 × 10^−11^	3.0199 × 10^−11^	3.0199 × 10^−11^	3.0199 × 10^−11^	3.0199 × 10^−11^	3.0199 × 10^−11^	6.5261 × 10^−7^	3.0199 × 10^−11^	3.0199 × 10^−11^
F26	8.1200 × 10^−4^	8.9934 × 10^−11^	3.2651 × 10^−2^	3.0199 × 10^−11^	3.0199 × 10^−11^	1.0937 × 10^−10^	3.0199 × 10^−11^	3.0199 × 10^−11^	3.0199 × 10^−11^	3.0199 × 10^−11^	3.0199 × 10^−11^
F27	9.9186 × 10^−11^	4.1191 × 10^−1^	5.4941 × 10^−11^	3.0199 × 10^−11^	3.0199 × 10^−11^	8.1014 × 10^−10^	3.0199 × 10^−11^	8.1527 × 10^−11^	3.0199 × 10^−11^	3.0199 × 10^−11^	3.0199 × 10^−11^
F28	3.0199 × 10^−11^	3.0199 × 10^−11^	3.0199 × 10^−11^	3.0199 × 10^−11^	3.0199 × 10^−11^	3.0199 × 10^−11^	3.0199 × 10^−11^	3.0199 × 10^−11^	3.1573 × 10^−5^	3.0199 × 10^−11^	3.0199 × 10^−11^
F29	1.4294 × 10^−8^	1.2235 × 10^−1^	3.1589 × 10^−10^	3.0199 × 10^−11^	3.0199 × 10^−11^	3.0199 × 10^−11^	3.0199 × 10^−11^	1.2057 × 10^−10^	8.6634 × 10^−5^	6.0658 × 10^−11^	6.0658 × 10^−11^
F30	3.0199 × 10^−11^	8.7663 × 10^−1^	3.0199 × 10^−11^	3.0199 × 10^−11^	3.0199 × 10^−11^	3.0199 × 10^−11^	3.0199 × 10^−11^	1.7666 × 10^−3^	3.0199 × 10^−11^	3.0199 × 10^−11^	4.6159 × 10^−10^

**Table 8:** The ranking of different algorithms on CEC2017.

Suites	CEC2017					
Dimensions	10		30		50	
Algorithms	M.R	T.R	M.R	T.R	M.R	T.R
PSO	3.03	2	2.57	2	2.50	2
DBO	5.83	6	5.60	5	5.60	4
DMO	4.73	3	5.53	4	6.10	6
IAO	5.13	4	6.90	8	8.37	10
MGO	12.00	12	11.93	12	11.93	12
PO	7.93	10	7.63	10	6.87	9
PLO	11.00	11	11.07	11	11.07	11
BKA	5.57	5	5.27	3	5.67	5
CPSOGSA	6.53	7	5.67	6	5.07	3
HPHHO	6.57	8	6.60	7	6.20	7
SCSO	7.33	9	6.90	9	6.23	8
MISCSO	**2.33**	**1**	**2.33**	**1**	**2.40**	**1**

**Table 9:** Parameters of each distributed power supply.

Type	Minimum Power/kW	Maximum Power/kW	$\mathrm{Operating}\;\mathrm{Costs}\;(\$\cdot {kg}^{-1})$	$\mathrm{Fuel}\;\mathrm{Costs}\;(\$\cdot {kg}^{-1})$
Photovoltaic Power (PV)	0	150	0.0450	0
Wind Power (WT)	0	100	0.0096	0
Energy Storage Device (BSS)	−60	60	0.0550	0
Diesel Generator (DE)	0	150	0.0780	0.21

**Table 10:** Pollutant treatment costs.

Pollutant Type	DE Emission Factor (kg/kWh)	$\mathrm{Governance}\;\mathrm{Expenses}\;(\$\cdot {kg}^{-1})$
${CO}_{2}$	4.33	0.028
${SO}_{2}$	0.46	5.950
${NO}_{2}$	2.32	8.510

**Table 11:** Unit price of electricity purchase and sale.

$\mathrm{Price}\;(\$\cdot h^{-1})$	Time Slot					
	Peak Hours		Off-Peak Hours		Regular Hours	
	10:00–14:00	18:00–20:00	07:00–09:00	15:00–17:00	00:00–06:00	23:00–24:00
Purchase electricity	0.69		0.46		0.31	
Electricity Sales	0.64		0.38		0.23	

**Table 12:** Cost results obtained from different algorithms.

Algorithm	Max	Min	Mean	Std	CPU Time	Rank
PSO	9053.31	5026.37	6571.94	679.16	213.96	2
DBO	9314.99	6153.18	6765.53	537.96	228.13	3
DMO	9922.43	6541.88	8371.58	598.43	235.05	8
IAO	13,126.44	10,707.35	11,052.59	412.19	246.11	12
MGO	9563.78	6098.10	7239.33	596.69	253.06	4
PO	8635.95	7569.52	8094.38	185.62	259.56	6
PLO	9862.89	7635.81	8639.25	394.87	265.03	10
BKA	9948.46	7991.50	8742.36	347.86	270.03	11
CPSOGSA	9849.87	6213.96	8234.95	623.58	288.50	7
HPHHO	9767.54	7036.79	8524.15	171.69	276.23	9
SCSO	9122.86	6584.94	7974.97	2097.3	208.54	5
MISCSO	6258.32	4986.87	5495.67	**242.91**	**210.08**	**1**

**Table 13:** Full forms of abbreviations commonly used in this article.

Abbreviation	Full Name
BKA	black-winged kite algorithm
CPSOGSA	hybridization of constriction coefficient based on particle swarm optimization and gravitational search algorithm
DBO	dung beetle optimizer
DMO	dwarf mongoose optimization algorithm
GOA	Grasshopper Optimization Algorithm
GWO	gray wolf optimizer
HPHHO	hybrid parallel Harris hawks optimization algorithm
IAO	information acquisition optimizer
IWO	Invasive Weed Optimization algorithm
MGO	moss growth optimization
MISCSO	Multi-Strategy Improved Sand Cat Swarm Optimization
PSO	particle swarm optimization
PO	parrot optimizer
PLO	polar lights optimizer
RVEA	real-valued evolutionary algorithm
RIME	Rime optimization algorithm
SCSO	sand cat optimization algorithm

**Table 14:** Common Symbols Used in This Article.

Symbol	Meaning
dim	Problem Dimensions
N	Population size
$P_{GRID}^{\iota }$	Interactive Power Between Microgrids and the Main Grid
$P_{PV}$	Photovoltaic power generation capacity
$P_{WT}$	Wind power generation capacity
$P_{DE}$	Diesel Generator Power
T	Maximum number of iterations

## Data Availability

Data are contained within the article.
